# Supplementary material for: Coenzyme Q deficiency causes impairment of the sulfide oxidation pathway
Source: EMBO Mol Med. 2016 Nov 17;9(1):96–111. doi: 10.15252/emmm.201606356 (PMC5210092; doi:10.15252/emmm.201606356)
Supplement: Supplementary file 1 — Appendix [file EMMM-9-96-s001.pdf]

Coenzyme Q deficiency causes impairment of the sulfide oxidation pathway

Marcello Ziosi[1]*, Ivano Di Meo[2] *, Giulio Kleiner[1], Xing-Huang Gao[3], Emanuele Barca[1,4], Maria J Sanchez- Quintero[1], Saba Tadesse[1] , Hongfeng Jiang[5], Changhong Qiao[5], Richard J Rodenburg[6], Emmanuel Scalais[7], Markus Schuelke[8], Belinda Willard[9], Maria Hatzoglou[3], Valeria Tiranti[2]# and Catarina M Quinzii[1]#

*These authors contributed equally to the work
#Co-last, co-corresponding authors

Appendix Table of contents:

Appendix Supplementary Methods

Appendix Figure Legends

Appendix Figures S1-S4

Appendix Tables S1-S2

Appendix supplementary methods

MitoSox

To estimate production of ROS, 80% confluent control and mutant skin fibroblasts cultured in DMEM medium were exposed to MitoSOX Red, a fluorochrome specific for anion superoxide produced in the inner mitochondrial compartment (Molecular Probes, Invitrogen Corp., Eugene, OR, USA) as previously described (Quinzii Faseb 2008; Quinzii Faseb 2010). Nucleai where stained with DAPI (0.1ug/ml) and MitoSOX intensity was monitored by fluorescence microscopy (Nikon Eclipse TE-2000). Images where elaborated and fluorescence intensity was measured with ImageJ.

Acylcarnitines determination

Sample preparation

All solvents for sample preparation and UPLC/MS/MS analysis were LC/MS grade (Fisher Scientific; Pittsburgh, PA). Free carnitine and fatty acylcarnitine standards were purchased from Toronto Research Chemicals (Toronto, Ontario, Canada) and R&D Systems, Inc. (Minneapolis, MN, USA). Deuterated internal standards were purchased from Cambridge Isotope Laboratories, Inc. (Tewksbury, MA, USA).

All the procedures were carried out on ice or at 4C. An aliquot of 100 µl methanol containing internal standards was added to 50 µl of plasma in a 1.5 ml microcentrifuge tube. After vortexing, 400 µl acetonitrile were added to the sample. The samples were vortexed at 2,000 rpm for 30 minutes and centrifuged at 20,000 x *g* for 10 min. The clear supernatant was transferred to an Agilent micro sampling vial (Agilent Technologies, Cat No 5184-3550, Santa Clara, CA, USA). The supernatant was evaporated to dryness with nitrogen at room temperature and subsequently reconstituted in 100 µl of 1:9 (v:v) methanol/water containing 10 mM ammonium acetate and 10 mM heptafluorobutyric acid for injection.

LC-MS Conditions

All experiments were carried out on an Agilent 6410 Triple Quad LC-MS/MS system. 2.5 µl are loaded onto an Agilent Poroshell 120 EC-C18 column (3.0 mm inner diameter × 50 mm with 2.7 µm particles). The column is maintained at 50°C throughout analysis. The initial conditions were as follows: 500 µl/min with a mobile phase composition of 98% solvent A (water containing 10 mM ammonium acetate and 10 mM heptafluorobutyric acid) and 2% solvent B (methanol containing 10 mM ammonium acetate and 10 mM heptafluorobutyric acid). Initial conditions are maintained for 1 minute. Solvent B is lineally increased to 90% over 5 minutes and maintained for 1 more minute. The column is cleaned with 98% solvent B for 3 minutes and conditioned with 2% solvent B for 3 minutes before the next injection. Carnitine and acylcarnitines of interest elute between 1.3 and 6.8 min. Positive electrospray tandem mass spectrometry (ESI-MS/MS) under multiple reactions monitoring mode (MRM) was performed employing the following parameters: capillary voltage, 2,000 V; gas temperature, 350 °C; gas flow, 13 l/min; nebulizer, 60 psi. Different species were identified by comparing the retention times of experimental compounds with those of authentic standards. Concentrations of carnitine and acylcarnitines in the serum were quantified by comparing integrated peak areas for those of each carnitines against those of known amounts of purified standards.

Mitochondrial respiratory chain enzyme activities in mouse tissues

Cytochrome oxidase (COX, complex IV), Citrate Synthase (CS) and Succinate Dehydrogenase (SDH, complex II) activities where measured by spectrophotometric assay. COX activity was measured following the oxidation of Cyt *C* catalyzed by cytochrome oxidase. Samples were incubated in medium containing 0.01 mM KH_2PO_4 (pH 7.0) and 1% reduced Cyt *C*, and the reaction followed for 3 min at 550 nm. The results were expressed in μmol oxidized Cyt *C*/min/mg protein. CS activity was measured following the reduction of 1 mM 5,5'-dithiobis(2-nitrobenzoic acid) in 1 M Tris–HCl (pH 8.1) at 412 nm for 2 min in the presence of 10 mM acetyl-CoA, and 10 mM oxalacetic acid. CS activity was expressed as nmol/min/g tissue. Succinate dehydrogenase activity was measured following reduction of 2,6 dichlorophenol-indophenol (DCIP) at 600 nm for 1 min. Enzymes activities were normalized to protein concentrations.

Appendix Supplementary figure legends

Appendix Fig. S1- Complex II-driven ADP-dependent respiration in fibroblasts: (**A**) Traces and (**B**) values of CIV-driven oxygen consumption in $3x10^6$ control (C) and 5 patient fibroblasts (P1-5). See Materials and Methods for the role and concentration of the compounds used.

Appendix Fig.S2- Complex IV-driven respiration in fibroblasts: (A) Representative traces and **(B)** values of CIV-driven oxygen consumption in $3x10^6$ control (C) and 5 patient fibroblasts (P1-5). See Materials and Methods for the role and concentration of the compounds used. Error bars represent SDs of two experiments.

Appendix Fig.S3- MitoSoX staining in COQ mutant fibroblasts: Relative MitoSox fluorescence intensity normalized to the background signal in control (C, N=2) and patients (P3, P4) fibroblasts. Error bars represent SDs of two experiments.

Appendix Fig.S4- NaSH treatment in control fibroblasts: **(A)** Representative western blots showing the level of SQR in two controls (C1 and C2) treated (+) and untreated (-) with NaSH for 24hrs. **(B)** Relative levels of SQR proteins normalized to Vinculin in

untreated controls and NaHS treated cells. **(C)** Relative levels of *SQR* transcripts normalized to *GAPDH.* Error bars represent SDs of 6 experiments.

Appendix Supplementary table legends

Appendix Table S1: Quantitation of cysteine containing peptides identified in the LC-MS/MS analysis of P1 and P4

Appendix Table S2: Acylcarnitines profile in blood of $Pdss2^{kd/kd}$ mice

Appendix Fig S1

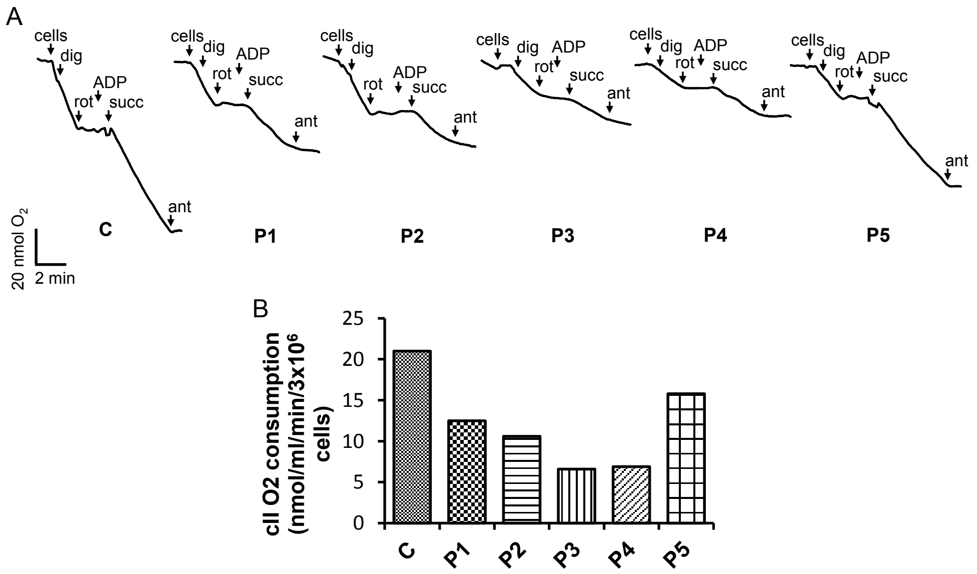

Appendix Fig S2

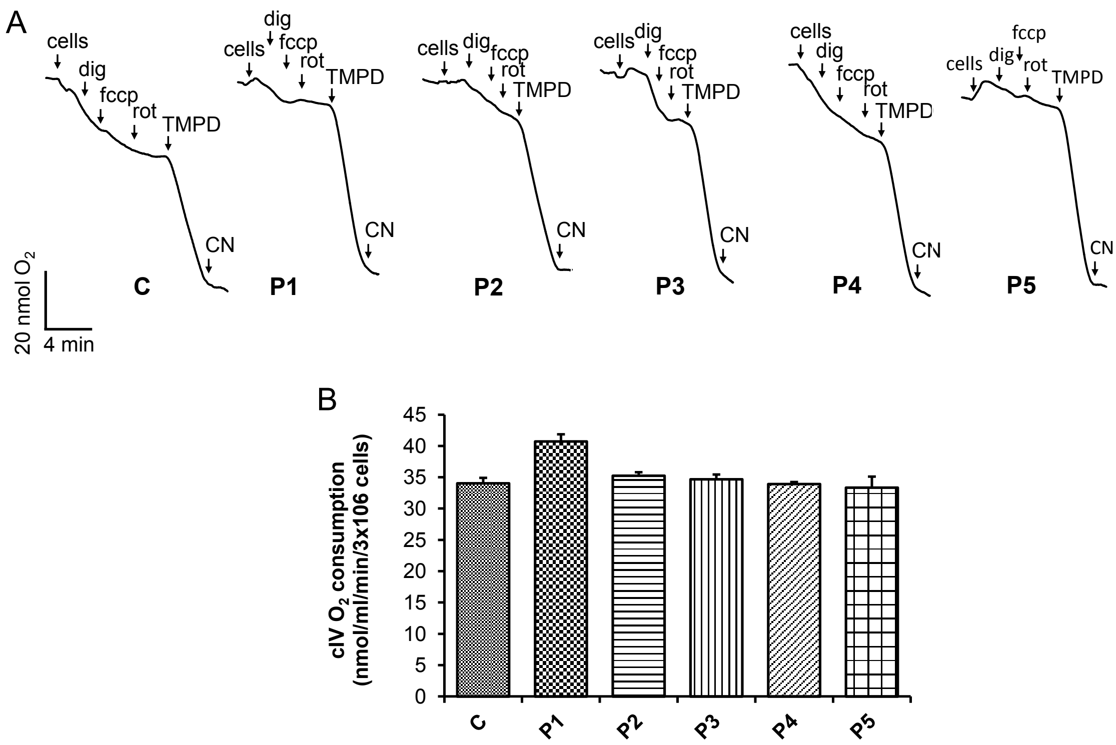

Appendix Fig S3

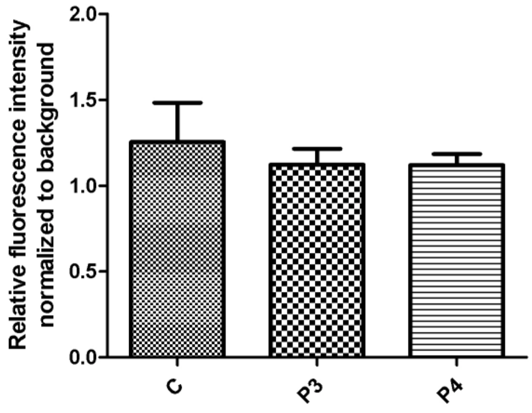

Appendix Fig S4

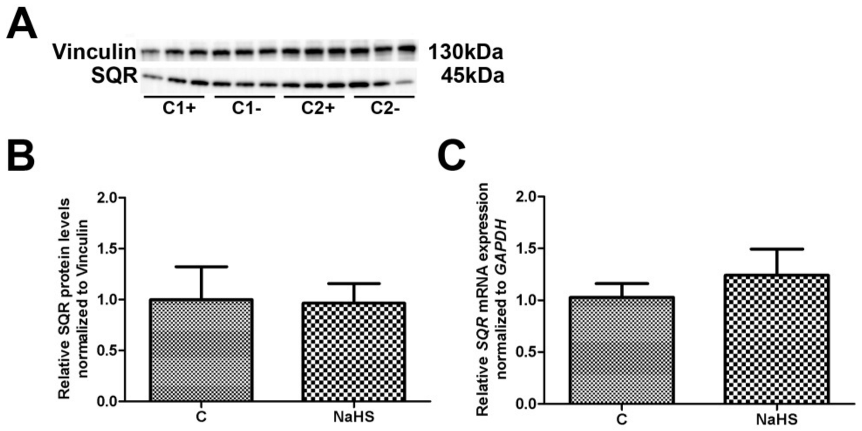

Appendix Table S1: Quantitation of cysteine containing peptides identified in the LC-MS/MS analysis of P1 and P4

Protein	Gene ID	Accession	[M+H]	Sequence	Site	H/L Ratios	
						P1	P4
14-3-3 protein gamma	YWHAG	P61981	1313.531	NcSETQYESK	C2	1.666	1.791
14-3-3 protein theta	YWHAQ	P27348	1202.454	YDDMATcMK	C7	1.321	1.341
14-3-3 protein theta	YWHAQ	P27348	1341.615	YLAEVAcGDDR	C7	1.262	2.184
14-3-3 protein theta	YWHAQ	P27348	1459.773	SIcTTVLELLDK	C3	1.274	1.637
14-3-3 protein theta	YWHAQ	P27348	1469.714	YLAEVAcGDDRK	C7	2.132	-
14-3-3 protein zeta/delta	YWHAZ	P63104	1177.474	YDDMAAcMK	C7	1.288	1.500
14-3-3 protein zeta/delta	YWHAZ	P63104	1491.778	DIcNDVLSLLEK	C3	1.909	1.312
26S protease regulatory subunit 7	PSMC2	P35998	1414.686	SVcTEAGMFAIR	C3	-	-
26S proteasome non-ATPase regulatory subunit 2	PSMD2	Q13200	1541.809	SGALLAcGIVNSGVR	C7	-	-
40S ribosomal protein S12	RPS12	P25398	1134.600	LGEWVGLcK	C8	-	-
40S ribosomal protein S12	RPS12	P25398	1824.009	LVEALcAEHQINLIK	C6	-	-
40S ribosomal protein S17	RPS17	P0CW22	1326.699	VcEEIAIIPSK	C2	1.400	1.928
40S ribosomal protein S2	RPS2	P15880	863.483	cGSVLVR	C1	1.659	1.789
40S ribosomal protein S2	RPS2	P15880	1212.612	GcTATLGNFAK	C2	1.442	2.169
40S ribosomal protein S21	RPS21	P63220	1092.550	TYAIcGAIR	C5	1.415	-
40S ribosomal protein S27	RPS27	P42677	1042.505	LTEGcSFR	C5	1.418	1.897
40S ribosomal protein S28	RPS28	P62857	1289.591	TGSQGQcTQVR	C7	1.344	1.902
40S ribosomal protein S29	RPS29	P62273	986.460	YGLNMcR	C6	3.327	-
40S ribosomal protein S3	RPS3	P23396	1361.727	GLcAIAQAESLR	C3	-	-
40S ribosomal protein S3a	RPS3A	P61247	1651.776	NcLTNFHGMDLTR	C2	-	2.849
40S ribosomal protein S3a	RPS3A	P61247	1772.879	AcQSIYPLHDVFVR	C2	1.437	-
40S ribosomal protein S4, X isoform	RPS4X	P62701	1341.761	EcLPLIIFLR	C2	2.033	1.590
40S ribosomal protein S4, X isoform	RPS4X	P62701	1855.891	FDTGNLcMVTGGANLGR	C7	1.615	-
40S ribosomal protein S5	RPS5	P46782	1574.854	VNQAIWLLcTGAR	C9	1.431	2.024
40S ribosomal protein S5	RPS5	P46782	1699.861	TIAEcLADELINAAK	C5	1.309	-
40S ribosomal protein S6	RPS6	P62753	1408.730	LNISFPATGcQK	C10	1.677	-
40S ribosomal protein S8	RPS8	P62241	1523.797	NcIVLIDSTPYR	C2	-	-
40S ribosomal protein SA	RPSA	P08865	1379.706	YVDIAIPcNNK	C8	1.895	-
40S ribosomal protein SA	RPSA	P08865	3064.508	ADHQPLTEASYVNLPTIALcNTDSPLR	C20	1.609	1.686
4F2 cell-surface antigen heavy chain	SLC3A2	P08195	1353.647	WcSWSLSQAR	C2	0.858	3.424
4-trimethylaminobutyraldehyde dehydrogenase	ALDH9A1	P49189	941.412	DDMTcVK	C5	1.744	1.460
4-trimethylaminobutyraldehyde dehydrogenase	ALDH9A1	P49189	1285.652	VIATFTcSGEK	C7	2.320	3.331
5'-nucleotidase	NT5E	P21589	823.397	cMGGVAR	C1	0.515	-
60 kDa heat shock protein, mitochondrial	HSPD1	P10809	1752.932	AAVEEGIVLGGGcALLR	C13	1.228	2.894
60S acidic ribosomal protein P0	RPLP0	P05388	1339.677	cFIVGADNVGSK	C1	4.183	-

60S acidic ribosomal protein P1	RPLP1	P05386	3882.008	ALANVNIGSLIcNVGAGGPAPAAGAAPAGGPAPSTAAAPAEEK	C12	1.771	-
60S ribosomal protein L10	RPL10	P27635	1048.456	MLScAGADR	C4	-	-
60S ribosomal protein L12	RPL12	P30050	1486.721	cTGGEVGATSALAPK	C1	3.219	3.011
60S ribosomal protein L13a	RPL13A	P40429	1502.712	cEGINISGNFYR	C1	4.069	-
60S ribosomal protein L14	RPL14	P50914	1283.643	ALVDGPcTQVR	C7	1.412	1.572
60S ribosomal protein L14	RPL14	P50914	1341.697	cMQLTDFILK	C1	2.437	-
60S ribosomal protein L18	RPL18	Q07020	1189.641	GcGTVLLSGPR	C2	1.959	-
60S ribosomal protein L18a	RPL18A	Q02543	1528.749	DLTTAGAVTQcYR	C11	1.845	2.435
60S ribosomal protein L18a	RPL18A	Q02543	1675.808	SSGEIVYcGQVFEK	C8	-	-
60S ribosomal protein L23	RPL23	P62829	2038.070	ISLGLPVGAVINcADNTGAK	C13	1.935	1.988
60S ribosomal protein L24	RPL24	P83731	1262.613	VELcSFSGYK	C4	-	2.188
60S ribosomal protein L27a	RPL27A	P46776	1490.696	NQSFcPTVNLDK	C5	1.876	-
60S ribosomal protein L3	RPL3	P39023	911.483	YcQVIR	C2	-	2.265
60S ribosomal protein L3	RPL3	P39023	1310.684	VAcIGAWHPAR	C3	-	1.383
60S ribosomal protein L30	RPL30	P62888	1426.826	LVILANNcPALR	C8	2.268	1.567
60S ribosomal protein L30	RPL30	P62888	1830.970	VcTLAIIDPGDSDIIR	C2	1.870	1.318
60S ribosomal protein L30	RPL30	P62888	2087.999	TGVHHYSGNNIELGTAcGK	C17	-	-
60S ribosomal protein L32	RPL32	P62910	1321.691	ELEVLLMcNK	C8	-	-
60S ribosomal protein L32	RPL32	P62910	1538.733	SYcAEIAHNVSSK	C3	2.788	3.340
60S ribosomal protein L34	RPL34	P49207	1017.455	AYGGSMcAK	C7	1.702	2.059
60S ribosomal protein L35a	RPL35A	P18077	876.434	cAYVYK	C1	-	-
60S ribosomal protein L36	RPL36	Q9Y3U8	1300.604	EVcGFAPYER	C3	1.726	1.867
60S ribosomal protein L4	RPL4	P36578	1257.551	SGQGAFGNMcR	C10	1.741	1.506
60S ribosomal protein L4	RPL4	P36578	1384.638	FcIWTESAFR	C2	1.669	2.207
60S ribosomal protein L4	RPL4	P36578	1678.864	GPcIIYNEDNGIIK	C3	1.231	-
60S ribosomal protein L4	RPL4	P36578	2010.085	YAIcSALAASALPALVMSK	C4	1.271	2.330
60S ribosomal protein L7a	RPL7A	P62424	1773.838	TcTTVAFTQVNSEDK	C2	2.508	1.999
60S ribosomal protein L9	RPL9	P32969	1591.809	MRPGVAcSVSQAQK	C7	-	2.664
6-phosphogluconate dehydrogenase, decarboxylating	PGD	P52209	1419.600	SAVENcQDSWR	C6	1.789	0.952
6-phosphogluconolactonase	PGLS	O95336	1251.546	WTLGFcDER	C6	-	0.781
6-phosphogluconolactonase	PGLS	O95336	1419.671	LcWFLDEAAAR	C2	-	-
72 kDa type IV collagenase	MMP2	P08253	1103.482	YEScTSAGR	C4	2.440	-
Acetyl-CoA acetyltransferase, cytosolic	ACAT2	Q9BWD1	2866.457	ATVAPEDVSEVIFGHVLAAGcGQNPVR	C21	2.049	-
Acidic leucine-rich nuclear phosphoprotein 32 family member A	ANP32A	P39687	2045.963	SLDLFNcEVTNLNDYR	C7	-	-
Aconitate hydratase, mitochondrial	ACO2	Q99798	1918.870	VGLIGScTNSSYEDMGR	C7	-	-
Actin, cytoplasmic 1	ACTB	P60709	850.415	cDVDIR	C1	1.652	-
Actin, cytoplasmic 1	ACTB	P60709	2618.198	LcYVALDFEQEMATAASSSSLEK	C2	1.630	1.636
Actin, gamma-enteric smooth muscle	ACTG2	P63267	3261.542	cPETLFQPSFIGMESAGIHETTYNSIMK	C1	-	-
Actin-related protein 2	ACTR2	P61160	1367.708	VVVcDNGTGFVK	C4	1.305	0.915
Actin-related protein 2/3	ARPC1A	Q92747	964.493	IVTcGADR	C4	1.162	-

complex subunit 1A							
Actin-related protein 2/3 complex subunit 2	ARPC2	O15144	1174.563	NcFASVFEK	C2	1.488	1.426
Actin-related protein 3	ACTR3	P61158	1316.661	YSYVcPDLVK	C5	1.791	-
Actin-related protein 3	ACTR3	P61158	1411.663	DYEEIGPSIcR	C10	-	-
Actin-related protein 3	ACTR3	P61158	3126.601	TLTGTVIDSGD GVTHVIPVAE GYVIGScIK	C28	-	0.922
Adenosine kinase	ADK	P55263	1481.780	SLIANLAAANc YK	C11	0.560	-
Adenylyl cyclase-associated protein 1	CAP1	Q01518	1224.574	TDGcHAYLSK	C4	0.906	-
Adenylyl cyclase-associated protein 1	CAP1	Q01518	1236.630	INSITVDNcK	C9	-	-
Adenylyl cyclase-associated protein 1	CAP1	Q01518	1308.653	NSLDcEIVSAK	C5	1.681	1.341
Adenylyl cyclase-associated protein 1	CAP1	Q01518	1830.944	ALLVTASQcQ QPAENK	C9	2.314	2.102
Adipocyte plasma membrane-associated protein	APMAP	Q9HDC9	852.445	SPFLcR	C5	1.614	-
ADP/ATP translocase 1	SLC25A4	P12235	1717.769	GADIMYTGTV DcWR	C12	-	-
ADP-ribosyl cyclase/cyclic ADP-ribose hydrolase 2	BST1	Q10588	1226.603	VADFLSWcR	C8	-	-
ADP-ribosylation factor 4	ARF4	P18085	1739.826	NIcFTVWDVG GQDR	C3	1.814	-
Alanine--tRNA ligase, cytoplasmic	AARS	P49588	3525.800	VVSIGVPVSEL LDDPSGPAGSL TSVEFcGGTHL R	C28	-	-
Alpha-2-HS-glycoprotein	AHSG	P02765	920.492	cNLLAEK	C1	-	-
Alpha-2-HS-glycoprotein	AHSG	P02765	1405.554	cDSSPDSAEDV R	C1	0.483	0.115
Alpha-2-HS-glycoprotein	AHSG	P02765	1533.648	cDSSPDSAEDV RK	C1	0.448	0.110
Alpha-actinin-1	ACTN1	P12814	1326.666	DGLGFcALIHR	C6	1.009	-
Alpha-actinin-1	ACTN1	P12814	1734.856	IcDQWDNLGA LTQK	C2	1.712	-
Alpha-actinin-1	ACTN1	P12814	2065.977	MVSDINNAWG cLEQVEK	C11	-	-
Alpha-actinin-4	ACTN4	O43707	1247.663	EGLLLWcQR	C7	-	-
Alpha-actinin-4	ACTN4	O43707	1465.707	TFTAWcNSHL R	C6	-	-
Alpha-actinin-4	ACTN4	O43707	1629.759	ELPPDQAEYcI AR	C10	-	-
Alpha-actinin-4	ACTN4	O43707	1681.866	cQLEINFNTLQ TK	C1	-	-
Alpha-actinin-4	ACTN4	O43707	1697.827	AcLISLGYDVE NDR	C2	2.026	2.129
Alpha-actinin-4	ACTN4	O43707	1826.849	IcDQWDALGSL THSR	C2	1.449	-
Aminopeptidase B	RNPEP	Q9H4A4	1725.900	LDSHPcLEVTA AALR	C6	0.856	0.655
Annexin A1	ANXA1	P04083	877.523	cLTAIVK	C1	1.452	1.342
Annexin A1	ANXA1	P04083	989.551	ILVALcGGN	C6	0.892	1.064
Annexin A1	ANXA1	P04083	1809.875	MYGISLcQAIL DETK	C7	0.961	-
Annexin A5	ANXA5	P08758	1186.569	ALLLLcGEDD	C6	1.051	1.137
Annexin A6	ANXA6	P08133	1091.547	ALLALcGGED	C6	-	0.541
Annexin A6	ANXA6	P08133	1114.580	FMTILcTR	C6	1.163	1.565
Annexin A6	ANXA6	P08133	1142.625	cLIEILASR	C1	2.176	0.748
Annexin A7	ANXA7	P20073	1250.641	LLVSMcQGNR	C6	1.378	2.046
Annexin A7	ANXA7	P20073	1800.856	LGTDEScFNMI LATR	C7	-	-
AP-2 complex subunit alpha-1	AP2A1	O95782	1371.646	AADLLYAMcD R	C9	1.411	1.451
AP-2 complex subunit beta	AP2B1	P63010	1060.474	EAQSIcER	C6	1.372	-
AP-2 complex subunit beta	AP2B1	P63010	1301.625	DcEDPNPLIR	C2	1.122	-
AP-2 complex subunit beta	AP2B1	P63010	1366.712	ITEYLcEPLR	C6	-	-
AP-2 complex subunit beta	AP2B1	P63010	1463.823	cVSTLLDLIQT K	C1	1.698	-
Asparagine--tRNA ligase,	NARS	O43776	1709.808	YGTcPHGGYG	C4	-	-

cytoplasmic				LGLER			
Asparagine--tRNA ligase, cytoplasmic	NARS	O43776	1756.900	LMTDTINEPILLcR	C13	1.298	-
Aspartate aminotransferase, mitochondrial	GOT2	P00505	1719.801	TcGFDFTGAVEDISK	C2	-	-
ATP-citrate synthase	ACLY	P53396	909.389	NcGSFTR	C2	1.498	1.343
ATP-citrate synthase	ACLY	P53396	1228.596	VDATADYIcK	C9	-	-
ATP-citrate synthase	ACLY	P53396	1383.711	FIcTTSAIQNR	C3	1.761	-
ATP-citrate synthase	ACLY	P53396	1733.799	AVQGMLDFDYVcSR	C12	-	-
ATP-dependent 6-phosphofructokinase, liver type	PFKL	P17858	1629.831	VFANAPDSAcVIGLK	C10	-	-
ATP-dependent 6-phosphofructokinase, muscle type	PFKM	P08237	1710.835	IFANTPDSGcVLGMR	C10	-	-
ATP-dependent 6-phosphofructokinase, platelet type	PFKP	Q01813	1018.553	AAcNLLQR	C3	-	-
ATP-dependent 6-phosphofructokinase, platelet type	PFKP	Q01813	2243.138	TNcNVAVINVGAPAAGMNAAVR	C3	0.826	-
ATP-dependent RNA helicase A	DHX9	Q08211	1430.748	AAEcNIVVTQPR	C4	1.481	-
ATP-dependent RNA helicase DDX1	DDX1	Q92499	1238.662	FLIcTDVAAR	C4	-	-
ATP-dependent RNA helicase DDX3Y	DDX3Y	O15523	1961.043	VRPcVVYGGADIGQQIR	C4	0.925	2.239
BAG family molecular chaperone regulator 3	BAG3	O95817	2267.975	SQSPAASDcSSSSSSASLPSSGR	C9	1.991	-
Basement membrane-specific heparan sulfate proteoglycan core protein	HSPG2	P98160	1103.468	TDEANcPTK	C6	0.998	1.596
Basement membrane-specific heparan sulfate proteoglycan core protein	HSPG2	P98160	1243.580	TcESLGAGGYR	C2	-	-
Basement membrane-specific heparan sulfate proteoglycan core protein	HSPG2	P98160	1426.700	TPSGLYLGTcER	C10	-	-
Beta-centractin	ACTR1B	P42025	1266.657	AcYLSINPQK	C2	-	-
Bifunctional purine biosynthesis protein PURH	ATIC	P31939	1382.754	VVAcNLYPFVK	C4	0.725	-
Calcyclin-binding protein	CACYBP	Q9HB71	1163.651	TDTVLILcR	C8	1.287	-
Calpain small subunit 1	CAPNS1	P04632	1214.551	TDGFGIDTcR	C9	1.418	1.060
Calpain-1 catalytic subunit	CAPN1	P07384	1149.576	cLQSGTLFR	C1	2.298	0.752
Calpain-1 catalytic subunit	CAPN1	P07384	1459.792	LEIcNLTPDALK	C4	-	-
Calpain-2 catalytic subunit	CAPN2	P17655	2057.017	LEIcNLTPDTLTSDTYK	C4	-	0.997
Calpastatin	CAST	P20810	1371.710	AAAPAPVSEAVcR	C12	-	-
Calpastatin	CAST	P20810	1643.695	cGEDDETIPSEYR	C1	-	-
Calponin-2	CNN2	Q99439	1403.639	cASQVGMTAPGTR	C1	1.324	1.152
Calponin-2	CNN2	Q99439	1462.644	cASQSGMTAYGTR	C1	1.654	1.488
Calponin-2	CNN2	Q99439	1549.791	AGQcVIGLQMGTNK	C4	-	0.799
Calponin-3	CNN3	Q15417	1446.651	cASQAGMTAYGTR	C1	-	-
Calreticulin	CALR	P27797	1549.713	HEQNIDcGGGYVK	C7	1.580	1.695
Catalase	CAT	P04040	1885.978	LGPNYLHIPVNcPYR	C12	1.358	3.188
Catechol O-methyltransferase	COMT	P21964	1878.015	YLPDTLLLEEcGLLR	C11	1.953	1.163
Catechol O-methyltransferase	COMT	P21964	1901.021	LITIEINPDcAAITQR	C10	-	-
Catenin alpha-1	CTNNA1	P35221	1104.587	IVAEcNAVR	C5	1.832	2.198
Cathepsin B	CTSB	P07858	1354.647	EQWPQcPTIK	C6	0.776	1.294
Cathepsin B	CTSB	P07858	1387.664	IcEPGYSPTYK	C2	-	1.291
Cathepsin B	CTSB	P07858	1891.900	GQDHcGIESEVVAGIPR	C5	0.944	1.590

Cathepsin L1	CTSL	P07711	1520.722	NHcGIASAASY PTV	C3	1.317	-
Cation-dependent mannose-6-phosphate receptor	M6PR	P20645	1117.546	AVVMIScNR	C7	2.332	-
CD151 antigen	CD151	P48509	1076.594	TVVALcGQR	C6	-	-
CD166 antigen	ALCAM	Q13740	1747.911	QIGDALPVScTI SASR	C10	-	-
CD44 antigen	CD44	P16070	1226.627	ALSIGFETcR	C9	2.231	1.410
CD59 glycoprotein	CD59	P13987	1607.694	FEHcNFNDVTT R	C4	1.035	1.209
CD81 antigen	CD81	P60033	1845.921	NNLcPSGSNIIS NLFK	C4	1.035	2.354
Cell cycle and apoptosis regulator protein 2	CCAR2	Q8N163	1353.699	VVTQNIcQYR	C7	0.943	3.188
Cellular nucleic acid-binding protein	CNBP	P62633	1059.505	cGESGHLAR	C1	1.818	2.372
Cellular nucleic acid-binding protein	CNBP	P62633	1101.504	TSEVNcYR	C6	1.869	1.482
Chloride intracellular channel protein 1	CLIC1	O00299	1151.568	IGNcPFSQR	C4	1.321	-
Chloride intracellular channel protein 1	CLIC1	O00299	1645.869	IEEFLEAVLcPP R	C10	1.106	-
Chloride intracellular channel protein 1	CLIC1	O00299	2001.007	FLDGNELTLA DcNLLPK	C12	1.455	-
Chloride intracellular channel protein 4	CLIC4	Q9Y696	1381.558	DEFTNTcPSDK	C7	1.444	2.203
Chloride intracellular channel protein 4	CLIC4	Q9Y696	1749.752	AGSDGESIGNc PFSQR	C11	1.296	1.203
Chloride intracellular channel protein 4	CLIC4	Q9Y696	2023.990	FLDGNEMTLA DcNLLPK	C12	2.870	-
Chloride intracellular channel protein 5	CLIC5	Q9NZA1	1780.846	AGIDGESIGNc PFSQR	C11	1.348	-
Clathrin heavy chain 1	CLTC	Q00610	1142.605	AHIAQLcEK	C7	1.175	-
Clathrin heavy chain 1	CLTC	Q00610	1237.480	ESNcYDPER	C4	0.981	1.619
Clathrin heavy chain 1	CLTC	Q00610	1398.648	DPHLAcVAYE R	C6	1.144	-
Clathrin heavy chain 1	CLTC	Q00610	1460.795	QNLQLcVQVA SK	C6	1.455	1.499
Clathrin heavy chain 1	CLTC	Q00610	1475.736	cNEPAVWSQL AK	C1	0.656	-
Clathrin heavy chain 1	CLTC	Q00610	1591.806	HSSLAGcQIIN YR	C7	1.154	1.307
Clathrin heavy chain 1	CLTC	Q00610	1628.913	LTDQLPLIIVcD R	C11	-	-
Coatomer subunit alpha	COPA	P53621	1496.822	LQLcYQLTTVG K	C4	2.140	-
Coatomer subunit alpha	COPA	P53621	1730.825	GHYNNVScAV FHPR	C8	-	-
Coatomer subunit alpha	COPA	P53621	2205.053	TTYQALPcLPS MYGYPNR	C8	1.840	2.807
Coatomer subunit beta'	COPB2	P35606	1063.579	VWcVASLR	C3	1.959	-
Coatomer subunit delta	ARCN1	P48444	893.494	ITLTcGR	C5	1.511	-
Coatomer subunit delta	ARCN1	P48444	1212.611	NYcNIQVTK	C3	0.937	-
Coatomer subunit gamma-1	COPG1	Q9Y678	1325.550	cVMDDDNEVR	C1	3.268	-
Coatomer subunit gamma-1	COPG1	Q9Y678	2095.097	SLPYNQPGTcY TLVALPK	C10	-	-
Cofilin-1	CFL1	P23528	1254.647	AVLFcLSEDK	C5	-	1.953
Cofilin-1	CFL1	P23528	1592.746	HELQANcYEE VK	C7	-	-
Collagen alpha-1(I) chain	COL1A1	P02452	2152.997	SGEYWIDPNQ GcNLDAIK	C12	4.440	-
Collagen alpha-2(I) chain	COL1A2	P08123	1462.730	FTYTVLVDGcS K	C10	-	-
Collagen alpha-2(I) chain	COL1A2	P08123	3028.375	LSHPEWSSGY YWIDPNQGcT MDAIK	C19	-	-
Collagen alpha-2(VI) chain	COL6A2	P12110	2247.091	HESENLYSIAc DKPQQVR	C11	-	-
Connective tissue growth factor	CTGF	P29279	1258.631	LPSPDcPFPR	C6	-	-
Coronin-1B	CORO1B	Q9BR76	1592.740	VTWDSTFcAV NPK	C8	0.860	0.321
Coronin-1C	CORO1C	Q9ULV4	1223.539	DTIcNQDER	C4	1.049	1.178
Coronin-1C	CORO1C	Q9ULV4	1271.538	NDQcYDDIR	C4	1.382	-

Coronin-1C	CORO1C	Q9ULV4	1611.761	VTWDSSFcAV NPR	C8	1.002	1.157
C-type mannose receptor 2	MRC2	Q9UBG0	1536.726	cFQVQGQEPQS R	C1	2.648	-
C-type mannose receptor 2	MRC2	Q9UBG0	2335.129	ISYHGScPQGL ADSAWIPFR	C7	-	-
Cullin-associated NEDD8-dissociated protein 1	CAND1	Q86VP6	1368.736	TYIQcIAAISR	C5	-	-
Cysteine and glycine-rich protein 1	CSRP1	P21291	957.441	DGELYcK	C6	1.939	1.859
Cysteine and glycine-rich protein 1	CSRP1	P21291	1199.578	cSQAVYAAEK	C1	2.588	2.441
Cysteine and glycine-rich protein 2	CSRP2	Q16527	1172.530	cGDSVYAAEK	C1	1.769	-
Cysteine and glycine-rich protein 2	CSRP2	Q16527	1636.742	TVYHAEEVQc DGR	C10	1.625	1.321
Cysteine-rich protein 2	CRIP2	P06733	1701.850	VNQIGSVTESI QAcK	C14	1.422	0.853
Cysteine-rich protein 2	CRIP2	P52943	1797.850	ASSVTTFTGEP NTcPR	C14	3.008	2.115
Cytochrome b-c1 complex subunit 1, mitochondrial	UQCRC1	P31930	1391.650	LcTSATESEVA R	C2	1.526	-
Cytochrome b-c1 complex subunit 2, mitochondrial	UQCRC2	P22695	1639.796	NALANPLYcPD YR	C9	1.509	4.165
Cytoplasmic dynein 1 heavy chain 1	DYNC1H1	Q14204	1192.579	LQGATcNNNK	C6	1.030	0.922
Cytoplasmic dynein 1 heavy chain 1	DYNC1H1	Q14204	1281.631	VQYPQSQAcK	C9	1.783	-
Cytoplasmic dynein 1 heavy chain 1	DYNC1H1	Q14204	1529.754	cYLTMTQALE AR	C1	3.004	-
Cytoskeleton-associated protein 4	CKAP4	Q07065	2162.963	SSSSSSASAAA AAAAASSSASc SR	C22	1.382	0.571
Cytosol aminopeptidase	LAP3	P28838	843.416	AAVAAGcR	C7	0.773	-
Cytosol aminopeptidase	LAP3	P28838	1761.853	ADMGGAATIc SAIVSAAK	C10	-	-
D-3-phosphoglycerate dehydrogenase	PHGDH	O43175	2121.078	ALVDHENVISc PHLGASTK	C11	-	-
Decorin	DCN	P07585	1593.774	DFEPSLGPVcP FR	C10	-	3.059
Desmoplakin	DSP	P15924	1334.653	NQcTQVVQER	C3	-	-
Destrin	DSTN	P60981	1196.641	AVIFcLSADK	C5	0.400	-
Destrin	DSTN	P60981	1612.719	HEcQANGPED LNR	C3	0.968	0.860
Dihydrolipoyl dehydrogenase, mitochondrial	DLD	P09622	1597.796	VcHAHPTLSEA FR	C2	-	1.498
Dihydropyrimidinase-related protein 2	DPYSL2	Q16555	1688.823	GLYDGPVcEVS VTPK	C8	0.868	-
Dihydropyrimidinase-related protein 2	DPYSL2	Q16555	1888.987	AITIANQTNcPL YITK	C10	0.450	3.128
DNA-(apurinic or apyrimidinic site) lyase	APEX1	P27695	1292.646	IcSWNVDGLR	C2	-	-
Dolichyl-diphosphooligosaccharide--protein glycosyltransferase subunit 1	RPN1	P04843	1125.488	TEGSDLcDR	C7	-	-
Ectonucleotide pyrophosphatase/phosphodiesterase family member 1	ENPP1	P22413	1306.575	TDNSEScVHGK	C7	12.70 6	-
Electron transfer flavoprotein subunit alpha, mitochondrial	ETFA	P13804	1363.736	LGGEVScLVA GTK	C7	1.240	1.556
Elongation factor 1-alpha 1	EEF1A1	P68104	3068.462	SGDAAIVDMV PGKPMcVESFS DYPPLGR	C16	1.592	-
Elongation factor 1-alpha 1	EEF1A1	P68104	3094.631	DGNASGTTLL EALDcILPPTRP TDKPLR	C15	-	-
Elongation factor 1-alpha 1	EEF1A1	P68104	3222.723	KDGNASGTTL LEALDcILPPTR PTDKPLR	C16	-	-
Elongation factor 1-gamma	EEF1G	P26641	1677.823	WFLTcINQPQF R	C5	1.392	1.657
Elongation factor 2	EEF2	P13639	1191.593	STLTDSLVcK	C9	-	1.250
Elongation factor 2	EEF2	P13639	1281.563	EGALcEENMR	C5	2.859	-
Elongation factor 2	EEF2	P13639	1451.775	cLYASVLTAQP R	C1	1.754	1.266
Elongation factor 2	EEF2	P13639	1507.713	DLEEDHAcIPIK	C8	1.273	1.152

Elongation factor 2	EEF2	P13639	1562.829	TFcQLILDPIFK	C3	2.099	1.734
Elongation factor 2	EEF2	P13639	1667.822	ETVSEESNVLcLSK	C11	2.015	-
Elongation factor 2	EEF2	P13639	2080.963	cELLYEGPPDDEAAMGIK	C1	2.570	2.498
Elongation factor 2	EEF2	P13639	2178.098	IWcFGPDGTGPNILTDITK	C3	1.933	1.349
Elongation factor 2	EEF2	P13639	2832.496	YVEPIEDVPcGNIVGLVGVDQFLVK	C10	1.718	1.424
Elongation factor 2	EEF2	P13639	3062.619	LMEPIYLVEIQcPEQVVGGIYGVLNR	C12	-	-
Endoplasmic reticulum resident protein 44	ERP44	Q9BS26	1516.686	VDcDQHSDIAQR	C3	1.884	-
Endoplasmin	HSP90B1	P14625	2429.133	LTESPcALVASQYGWSGNMER	C6	1.399	1.897
Endosialin	CD248	Q9HCU0	1151.588	GSLTGVQTcR	C9	-	-
Endosialin	CD248	Q9HCU0	1296.604	cVALEASGEHR	C1	-	-
Eukaryotic translation initiation factor 2 subunit 1	EIF2S1	P05198	1507.731	AGLNcSTENMPIK	C5	1.584	-
Eukaryotic translation initiation factor 2 subunit 3	EIF2S3	P41091	1968.889	ScGSSTPDEFPTDIPGTK	C2	-	-
Eukaryotic translation initiation factor 3 subunit I	EIF3I	Q13347	1389.662	HVLTGSADNScR	C11	-	-
Eukaryotic translation initiation factor 6	EIF6	P56537	2616.317	LSALGNVTTcNDYVALVHPDLDR	C10	1.620	-
Extended synaptotagmin-1	ESYT1	Q9BSJ8	1137.536	LGTQTFcSR	C7	1.196	0.840
Extracellular matrix protein 1	ECM1	Q16610	1373.690	LTFINDLcGPR	C8	-	-
Far upstream element-binding protein 1	FUBP1	Q96AE4	1612.850	cQHAAEIITDLLR	C1	2.745	-
Fascin	FSCN1	Q16658	1018.426	DGNVTcER	C6	1.648	1.826
Fascin	FSCN1	Q16658	1100.494	GEHGFIGcR	C8	1.429	-
Fascin	FSCN1	Q16658	1505.731	LScFAQTVSPAEK	C3	2.104	-
Fascin	FSCN1	Q16658	1684.801	YLAADKDGNVTcER	C12	1.855	-
Fatty acid synthase	FASN	P49327	1481.784	LSIPTYGLQcTR	C10	-	1.637
Fatty acid synthase	FASN	P49327	2740.387	cVLLSNLSSTSHVPEVDPGSAELQK	C1	-	-
Fibrillin-1	FBN1	P35555	879.441	VGcVDTR	C3	-	3.824
Fibrillin-1	FBN1	P35555	1258.554	FTSTGQcNDR	C7	1.684	4.882
Fibrillin-1	FBN1	P35555	1348.696	cSNQLPQSITK	C1	-	-
Fibronectin	FN1	P02751	1069.498	cTcVGNGR	C1; C3	2.565	2.191
Fibronectin	FN1	P02751	1294.580	TFYScTTEGR	C5	1.595	2.413
Fibronectin	FN1	P02751	1556.760	GEWTcIAYSQLR	C5	1.888	2.639
Fibronectin	FN1	P02751	1608.805	GNLLQcIcTGNGR	C6; C8	-	-
Fibronectin	FN1	P02751	1654.681	WcGTTQNYDADQK	C2	1.469	2.510
Fibronectin	FN1	P02751	1713.686	cDPHEATcYDDGK	C1; C8	-	-
Fibronectin	FN1	P02751	1787.797	DSMIWDcTcIGAGR	C7; C9	-	3.047
Fibronectin	FN1	P02751	1799.885	YSFcTDHTVLVQTR	C4	1.448	2.333
Fibronectin	FN1	P02751	1927.874	TYLGNALVcTcYGGSR	C9; C11	1.104	2.640
Fibronectin	FN1	P02751	2097.960	EYLGAIcScTcFGGQR	C7; C9; C11	-	-
Fibulin-2	FBLN2	P98095	911.478	NPLIcAR	C5	0.994	-
Fibulin-2	FBLN2	P98095	1066.543	ScMAGVLGAK	C2	-	-
Fibulin-2	FBLN2	P98095	2312.152	TcRPEGHPPQPEAPQEPALK	C2	-	-
Filamin-A	FLNA	P21333	938.405	GDGScDVR	C5	-	-
Filamin-A	FLNA	P21333	943.431	cSGPGLER	C1	1.540	0.936

Filamin-A	FLNA	P21333	1103.507	SSFTVDcSK	C7	1.941	-
Filamin-A	FLNA	P21333	1161.625	TPcEEILVK	C3	1.212	1.300
Filamin-A	FLNA	P21333	1223.645	IVGPSGAAVPcK	C11	1.559	-
Filamin-A	FLNA	P21333	1290.634	cSGPGLSPGMVR	C1	2.346	2.228
Filamin-A	FLNA	P21333	1303.655	AHVVPcFDASK	C6	-	1.381
Filamin-A	FLNA	P21333	1657.690	DNGNGTYScSYVPR	C9	1.170	1.544
Filamin-A	FLNA	P21333	1709.770	VDINTEDLEDGTcR	C13	-	-
Filamin-A	FLNA	P21333	1852.913	VQVQDNEGcPVEALVK	C9	1.656	2.343
Filamin-A	FLNA	P21333	1862.885	ATcAPQHGAPGPGPADASK	C3	1.582	1.528
Filamin-A	FLNA	P21333	1955.000	VTYcPTEPGNYIINIK	C4	1.169	1.795
Filamin-A	FLNA	P21333	2088.049	cAPGVVGPAEADIDFDIIR	C1	2.144	-
Filamin-A	FLNA	P21333	2125.956	THEAEIVEGENHTYcIR	C15	1.760	-
Filamin-A	FLNA	P21333	2331.086	DGScGVAYVVQEPGDYEVSVK	C4	-	-
Filamin-A	FLNA	P21333	2350.089	VHSPSGALEEcYVTEIDQDK	C11	-	-
Filamin-A	FLNA	P21333	2609.209	YWPQEAGEYAVHVLcNSEDIR	C15	-	0.812
Filamin-A	FLNA	P21333	2756.374	cSYQPTMEGVHTVHVTFAGVPIPR	C1	1.708	1.965
Filamin-A	FLNA	P21333	2836.401	LQVEPAVDTSGVQcYGPGIEGQGVFR	C14	1.512	1.224
Filamin-B	FLNB	O75369	1271.607	VAVTEGcQPSR	C7	0.606	-
Filamin-B	FLNB	O75369	1565.710	MDGTYAcSYTPVK	C7	-	-
Filamin-B	FLNB	O75369	1690.750	VDIQTEDLEDGTcK	C13	0.504	-
Filamin-B	FLNB	O75369	1712.890	APSVATVGSIcDLNLK	C11	0.718	-
Filamin-B	FLNB	O75369	1729.744	IEYNDQNDGScDVK	C11	-	-
Filamin-B	FLNB	O75369	1746.873	GAGTGGLGLTVEGPcEAK	C15	-	-
Filamin-C	FLNC	Q14315	1098.537	cSGPGLGAGVR	C1	1.224	1.316
Filamin-C	FLNC	Q14315	1197.587	TPcEEVYVK	C3	-	-
Filamin-C	FLNC	Q14315	1263.603	VGVTEGcDPTR	C7	-	1.176
Filamin-C	FLNC	Q14315	1265.698	cSYVPTKPIK	C1	0.696	1.083
Filamin-C	FLNC	Q14315	1387.693	APSGNEEPcLLK	C9	1.930	1.518
Filamin-C	FLNC	Q14315	1638.797	VPQTFTVDcSQAGR	C9	0.890	0.927
Filamin-C	FLNC	Q14315	1651.806	LYAQDADGcPIDIK	C9	0.792	-
Filamin-C	FLNC	Q14315	1856.945	QAPSIATIGSTcDLNLK	C12	0.953	1.184
Filamin-C	FLNC	Q14315	1865.937	ARVPQTFTVDcSQAGR	C11	0.802	-
Filamin-C	FLNC	Q14315	1936.976	VTYcPTEPGTYIINIK	C4	0.731	0.448
Filamin-C	FLNC	Q14315	2077.917	SPFPVHVSEAcNPNAcR	C11; C16	-	1.115
Filamin-C	FLNC	Q14315	2083.930	EAGDGVFEcEYYPVVPGK	C9	1.271	0.638
Filamin-C	FLNC	Q14315	2295.050	VHTPSGAVEEcYVSELDSDK	C11	1.591	-
Filamin-C	FLNC	Q14315	2356.069	DGScGVSYVVQEPGDYEVSIK	C4	0.987	-
Filamin-C	FLNC	Q14315	2358.169	DGTcTVSYLPTAPGDYSIIVR	C4	0.924	1.551
Filamin-C	FLNC	Q14315	2628.280	EcPEGHVVTYTPMAPGNYLIAIK	C2	-	-

Filamin-C	FLNC	Q14315	2712.357	cTYRPAMEGPHTVHVAFAGAPITR	C1	0.901	1.125
Four and a half LIM domains protein 1	FHL1	Q13642	1241.557	FWHDTcFR	C6	-	3.392
Four and a half LIM domains protein 1	FHL1	Q13642	1572.826	cLHPLANETFVAK	C1	-	-
Four and a half LIM domains protein 2	FHL2	Q14192	964.530	cSLSLVGR	C1	0.881	-
Four and a half LIM domains protein 2	FHL2	Q14192	1796.791	FDcHHcNESLFGK	C3; C6	-	1.011
Fructose-bisphosphate aldolase A	ALDOA	P04075	1000.482	cQYVTEK	C1	1.509	1.068
Fructose-bisphosphate aldolase A	ALDOA	P04075	1200.607	ALANSLAcQGK	C8	1.100	1.145
Fructose-bisphosphate aldolase A	ALDOA	P04075	2161.151	VNPcIGGVILFHETLYQK	C4	-	-
Fructose-bisphosphate aldolase A	ALDOA	P04075	3093.560	YASIcQQNGIVPIVEPEILPDGDHDLK	C5	1.157	0.965
Galectin-1	LGALS1	P09382	1554.712	DSNNLcLHFNPR	C6	1.595	0.721
Galectin-1	LGALS1	P09382	1715.779	FNAHGDANTIVcNSK	C12	1.407	1.202
Galectin-1	LGALS1	P09382	2935.457	EAVFPFQPGSVAEVcITFDQANLTVK	C15	-	-
GDP-fucose protein O-fucosyltransferase 1	POFUT1	Q9H488	1386.691	VAYcFEVAAQR	C4	1.105	0.738
Glucosamine 6-phosphate N-acetyltransferase	GNPNAT1	Q96EK6	1374.622	VEDVVVSDEcR	C10	-	-
Glucosidase 2 subunit beta	PRKCSH	P14314	1625.719	YEQGTGcWQGPNR	C7	1.467	1.491
Glucosylceramidase	GBA	P04062	1355.645	VPMAScDFSIR	C6	-	-
Glutathione S-transferase P	GSTP1	P09211	1204.604	AScLYGQLPK	C3	1.105	1.854
Glyceraldehyde-3-phosphate dehydrogenase	GAPDH	P04406	1603.853	VPTANVSVVDLTcR	C13	-	0.706
Glyceraldehyde-3-phosphate dehydrogenase	GAPDH	P04406	1969.969	IISNAScTTNcLAPLAK	C7; C11	-	-
Glycine--tRNA ligase	GARS	P41250	1699.817	TSYGWIEIVGcADR	C11	2.517	1.301
Glycine--tRNA ligase	GARS	P41250	2097.061	cSVLPLSQNQEFMPFVK	C1	1.477	-
Glycogen phosphorylase, brain form	PYGB	P11216	1915.047	WLLLcNPGLADTIVEK	C5	-	-
Golgi apparatus protein 1	GLG1	Q92896	1074.542	cNVENLPR	C1	2.267	-
Golgi apparatus protein 1	GLG1	Q92896	1295.600	LSSDcEDQIR	C5	1.358	0.480
Golgi apparatus protein 1	GLG1	Q92896	1341.590	FcENTQAGEGR	C2	2.343	2.481
Guanine nucleotide-binding protein G(i) subunit alpha-2	GNAI2	P04899	1183.583	LFDSIcNNK	C6	-	1.307
Guanine nucleotide-binding protein G(i) subunit alpha-2	GNAI2	P04899	1589.768	LWADHGVQAcFGR	C10	-	-
Guanine nucleotide-binding protein G(i) subunit alpha-2	GNAI2	P04899	2117.039	ITHSPLTIcFPEYTGANK	C9	1.135	-
Guanine nucleotide-binding protein G(I)/G(S)/G(T) subunit beta-1	GNB1	P62873	1298.647	LFVSGAcDASAK	C7	-	-
Guanine nucleotide-binding protein G(I)/G(S)/G(T) subunit beta-1	GNB1	P62873	2084.013	AcADATLSQITNNIDPVGR	C2	1.930	1.404
Guanine nucleotide-binding protein G(I)/G(S)/G(T) subunit beta-1	GNB1	P62873	2489.189	VScLGVTDDGMAVATGSWDSFLK	C3	-	-
Guanine nucleotide-binding protein G(I)/G(S)/G(T) subunit beta-2	GNB2	P62879	1323.628	TFVSGAcDASIK	C7	1.412	1.839
Guanine nucleotide-binding protein G(I)/G(S)/G(T) subunit beta-2	GNB2	P62879	1999.979	AcGDSTLTQITAGLDPVGR	C2	-	-
Guanine nucleotide-binding protein G(k) subunit alpha	GNAI3	P08754	1169.542	DGGVQAcFSR	C7	-	-
Guanine nucleotide-binding protein subunit beta-4	GNB4	Q9HAV0	1297.574	TFVSGAcDASSK	C7	-	1.650
Heat shock 70 kDa protein 4	HSPA4	P34932	1454.720	FLEMcNDLLAR	C5	-	-
Heat shock 70 kDa protein 4	HSPA4	P34932	1721.876	SVMDATQIAGLNcLR	C13	2.488	0.822
Heat shock cognate 71 kDa	HSPA8	P11142	1012.550	VcNPIITK	C2	-	1.756

protein							
Heat shock cognate 71 kDa protein	HSPA8	P11142	1377.691	cNEIINWLDK	C1	1.262	-
Heat shock protein 105 kDa	HSPH1	Q92598	1701.940	SVLDAAQIVGLNcLR	C13	1.612	-
Heat shock protein beta-7	HSPB7	Q9UBY9	1960.970	cQLPEDVDPTSVTSALR	C1	0.457	-
Heterogeneous nuclear ribonucleoprotein A/B	HNRNPAB	Q99729	1240.628	FGEVVDcTLK	C7	0.539	-
Heterogeneous nuclear ribonucleoprotein A1-like 2	HNRNPA1L2	Q32P51	2038.943	SHFEQWGTLTDcVVMR	C12	-	-
Heterogeneous nuclear ribonucleoprotein A3	HNRNPA3	P51991	1410.692	WGTLTDcVVMR	C7	1.636	1.421
Heterogeneous nuclear ribonucleoprotein H	HNRNPH1	P31943	1577.745	GLPWScSADEVQR	C6	-	-
Heterogeneous nuclear ribonucleoprotein H2	HNRNPH2	P55795	1580.727	GLPWScSADEVMR	C6	3.230	-
Heterogeneous nuclear ribonucleoprotein U	HNRNPU	Q00839	1202.583	McLFAGFQR	C2	-	-
Heterogeneous nuclear ribonucleoproteins A2/B1	HNRNPA2B1	P22626	1066.544	LTDcVVMR	C4	-	-
Hexokinase-1	HK1	P19367	1529.747	AAQLcGAGMAAVVDK	C5	-	-
High mobility group protein B2	HMGB2	P26583	1569.723	MSSYAFFVQTcR	C11	-	-
Histidine triad nucleotide-binding protein 1	HINT1	P49773	2359.228	cLAFHDISPQAPTHFLVIPK	C1	1.267	1.266
HLA class I histocompatibility antigen, B-37 alpha chain	HLA-B	P18463	1569.777	AYLEGTcVEWLR	C7	-	2.864
Importin-5	IPO5	O00410	1185.596	VcDIAAELAR	C2	-	-
Importin-5	IPO5	O00410	1698.861	VAAAESMPLLLEcAR	C13	0.504	-
Inactive tyrosine-protein kinase 7	PTK7	Q13308	1454.712	LQDSGTFQcVAR	C9	-	-
Inosine-5'-monophosphate dehydrogenase 2	IMPDH2	P12268	1503.744	HGFcGIPITDTGR	C4	1.509	-
Integrin beta-1	ITGB1	P05556	1471.667	SNGLIcGGNGVcK	C6; C12	-	-
Integrin beta-1	ITGB1	P05556	1755.702	FcEcDNFNcDR	C2; C4; C9	-	-
Interphotoreceptor matrix proteoglycan 1	IMPG1	Q17R60	2509.177	CKPGYDSQGSLDGLEPGLcGPGTK	C19	-	-
Isoleucine--tRNA ligase, cytoplasmic	IARS	P41252	1235.594	VcMDFNIIR	C2	1.534	2.449
Keratin, type II cytoskeletal 1	KRT1	P04264	1833.758	FSScGGGGGSFGAGGGFGSR	C4	-	0.683
Lactotransferrin	LTF	P02788	1430.739	cGLVPVLAENYK	C1	-	-
Leucine-rich PPR motif-containing protein, mitochondrial	LRPPRC	P42704	1477.734	cVANNQVETLEK	C1	-	-
Leucine-rich repeat-containing protein 15	LRRC15	Q8TF66	1151.551	ASQVEcTGAR	C6	1.367	0.571
Leucine-rich repeat-containing protein 15	LRRC15	Q8TF66	1805.927	LGTDTVPVcFSPANVR	C9	0.365	-
Leupaxin	LPXN	O60711	2097.984	SGLAYcPNDYHQLFSPR	C6	-	0.699
LIM domain only protein 7	LMO7	Q8WWI1	1412.710	SLTScSSDITLR	C5	-	-
L-lactate dehydrogenase A chain	LDHA	P00338	1316.628	VIGSGcNLDSAR	C6	0.880	0.849
L-lactate dehydrogenase A chain	LDHA	P00338	2362.237	DDVFLSVPcILGQNGISDLVK	C9	0.836	0.931
Lupus La protein	SSB	P05455	1381.569	FSGDLDDQTcR	C10	-	-
Lysosome-associated membrane glycoprotein 1	LAMP1	P11279	1087.501	cNAEEHVR	C1	1.538	1.590
Macrophage migration inhibitory factor	MIF	P14174	1112.615	LLcGLLAER	C3	0.844	1.449
Major vault protein	MVP	Q14764	1371.640	HYcTVANPVSR	C3	1.110	1.269
Malate dehydrogenase, cytoplasmic	MDH1	P40925	1830.981	VIVVGNPANTNcLTASK	C12	-	-
Malate dehydrogenase, mitochondrial	MDH2	P40926	1221.581	EGVVEcSFVK	C6	1.616	-
Mesencephalic astrocyte-derived neurotrophic factor	MANF	P55145	1629.748	LcYYIGATDDAATK	C2	-	1.652

Methionine--tRNA ligase, cytoplasmic	MARS	P56192	1972.077	LFVSDGVPGcLPVLAAAGR	C10	1.739	-
Mitochondrial 10-formyltetrahydrofolate dehydrogenase	ALDH1L2	Q3SY69	1940.875	ScDVEPNDTVDALYNR	C2	-	-
Mitochondrial antiviral-signaling protein	MAVS	Q7Z434	1047.488	ATcTLSGNR	C3	-	1.358
Mitochondrial-processing peptidase subunit beta	PMPCB	O75439	1424.714	LcTSVTESEVAR	C2	1.719	-
Moesin	MSN	P26038	1793.885	ILALcMGNHELYMR	C5	-	-
Muscleblind-like protein 2	MBNL2	Q5VZF2	1022.474	ScQVENGR	C2	1.534	-
Myoferlin	MYOF	Q9NZM1	1027.468	cYVYQAR	C1	-	1.232
Myoferlin	MYOF	Q9NZM1	1161.559	VHVcGQTHR	C4	1.096	-
Myoferlin	MYOF	Q9NZM1	1602.788	ELPDSVPQEcTVR	C10	1.103	-
Myosin light chain kinase, smooth muscle	MYLK	Q15746	1066.577	FLLDcGIR	C5	-	-
Myosin light chain kinase, smooth muscle	MYLK	Q15746	1359.604	YTcEATNGSGAR	C3	1.740	-
Myosin light polypeptide 6	MYL6	P60660	1414.687	ILYSQcGDVMR	C6	1.137	-
Myosin-9	MYH9	P35579	1022.499	QSAcNLEK	C4	-	1.573
Myosin-9	MYH9	P35579	1081.511	cQHLQAEK	C1	2.063	2.340
Myosin-9	MYH9	P35579	1090.574	cNGVLEGIR	C1	2.233	1.570
Myosin-9	MYH9	P35579	1367.683	ADFcIIHYAGK	C4	-	1.326
Myosin-9	MYH9	P35579	1624.751	EDQSILcTGESGAGK	C7	1.532	1.396
Myosin-9	MYH9	P35579	1842.904	KQELEEIcHDLEAR	C8	-	-
Myosin-9	MYH9	P35579	2359.173	VEDMAELTcLNEASVLHNLK	C9	1.150	-
NADH dehydrogenase [ubiquinone] 1 beta subcomplex subunit 7	NDUFB7	P17568	1390.564	HDWDYcEHR	C6	-	2.623
NADH-cytochrome b5 reductase 3	CYB5R3	P00387	2213.036	DPDDHTVcHLLFANQTEK	C8	0.901	-
Nestin	NES	P48681	1300.684	VGLNAQAAcAPR	C9	-	0.630
Niban-like protein 1	FAM129B	Q96TA1	1343.606	HcNNGIPEDSK	C2	1.081	-
Non-POU domain-containing octamer-binding protein	NONO	Q15233	1321.674	FAcHSASLTVR	C3	1.859	1.936
Nuclear transport factor 2	NUTF2	P61970	1828.823	NINDAWVcTNDMFR	C8	2.243	-
Oligosaccharyltransferase complex subunit OSTC	OSTC	Q9NRP0	1496.825	VPFLVLEcPNLK	C8	1.753	-
Palladin	PALLD	Q8WX93	1245.590	NEAGIVScTAR	C8	1.905	1.050
Palladin	PALLD	Q8WX93	1427.705	DAGIYTcIATNR	C7	1.387	2.209
PDZ and LIM domain protein 1	PDLIM1	O00151	1393.722	GcTDNLTLTVAR	C2	-	-
PDZ and LIM domain protein 4	PDLIM4	P50479	1213.678	cGHGIVGTIVK	C1	-	-
PDZ and LIM domain protein 4	PDLIM4	P50479	1741.934	LGAPLSGLQGLPEcTR	C14	1.738	-
PDZ and LIM domain protein 7	PDLIM7	Q9NR12	1718.721	AFYMEEGVPYcER	C11	-	0.491
Peptidyl-prolyl cis-trans isomerase A	PPIA	P62937	1192.595	ITIADcGQLE	C6	1.857	-
Peptidyl-prolyl cis-trans isomerase A	PPIA	P62937	1671.800	IIPGFMcQGGDFTR	C7	1.976	1.607
Peptidyl-prolyl cis-trans isomerase A	PPIA	P62937	2859.358	HTGPGILSMANAGPNTNGSQFFIcTAK	C24	0.836	1.387
Peptidyl-prolyl cis-trans isomerase B	PPIB	P23284	1063.549	DVIIADcGK	C7	-	-
Peptidyl-prolyl cis-trans isomerase FKBP10	FKBP10	Q96AY3	1593.727	GMDQGLLGMcPGER	C10	3.072	-
Peptidyl-prolyl cis-trans isomerase FKBP10	FKBP10	Q96AY3	1906.944	VIEGLDTGLQGMcVGER	C13	3.711	1.495
Peptidyl-prolyl cis-trans isomerase-like 1	PPIL1	Q9Y3C6	1206.612	VcQGIGMVNR	C2	-	2.070
Perilipin-3	PLIN3	O60664	2703.409	DIAQQLQATcTSLGSSIQGLPTNVK	C10	1.461	-
Peroxiredoxin-6	PRDX6	P30041	1468.716	DFTPVcTTELGR	C6	5.471	0.675

Peroxiredoxin-6	PRDX6	P30041	1650.695	DINAYNcEEPTEK	C7	-	-
Persulfide dioxygenase ETHE1, mitochondrial	ETHE1	O95571	1389.757	SLLPGcQSVISR	C6	0.739	-
Phosphatidylethanolamine-binding protein 1	PEBP1	P30086	1171.541	cDEPILSNR	C1	1.992	1.437
Phosphatidylethanolamine-binding protein 1	PEBP1	P30086	2137.957	APVAGTcYQAEWDDYVPK	C7	1.608	1.294
Phosphoglycerate kinase 1	PGK1	P00558	1841.997	AcANPAAGSVILLENLR	C2	1.005	1.221
Phosphoglycerate kinase 1	PGK1	P00558	1973.961	GcITIIGGGDTATccAK	C2; C14; C15	-	-
Phosphoglycerate mutase 1	PGAM1	P18669	1941.915	YADLTEDQLPScESLK	C12	0.995	1.263
Phosphomevalonate kinase	PMVK	Q15126	1146.638	LGADVcAVLR	C6	-	-
Phosphoserine aminotransferase	PSAT1	Q9Y617	1500.720	cADYVVTGAWSAK	C1	-	-
Plasma membrane calcium-transporting ATPase 4	ATP2B4	P23634	1481.643	EGDFGcTVMELR	C6	0.901	-
Plasma membrane calcium-transporting ATPase 4	ATP2B4	P23634	2218.112	DALTQINVHYGGVQNLcSR	C17	-	-
Plasminogen activator inhibitor 2	SERPINB2	P05120	1308.680	ITNcILFFGR	C4	-	-
Plastin-3	PLS3	P13797	1104.613	AVGDGIVLcK	C9	0.864	1.223
Platelet-activating factor acetylhydrolase IB subunit alpha	PAFAH1B1	P43034	2435.176	MVRPNQDGTLIAScSNDQTVR	C14	0.828	-
Plectin	PLEC	Q15149	891.477	LEcLQR	C3	2.878	-
Plectin	PLEC	Q15149	1068.552	VPLDVAcAR	C7	2.473	-
Plectin	PLEC	Q15149	1134.473	GcLDEETSR	C2	3.574	2.825
Plectin	PLEC	Q15149	1171.522	AFcGFEDPR	C3	3.679	-
Plectin	PLEC	Q15149	1248.595	cVEDPETGLR	C1	3.035	-
Plectin	PLEC	Q15149	1264.703	LPLLAVcDYK	C7	-	2.530
Plectin	PLEC	Q15149	2000.096	cRPDQLTGLSLLPLSEK	C1	4.819	-
Plectin	PLEC	Q15149	2012.041	TLLQGSGcLAGIYLEDTK	C8	3.072	-
Plectin	PLEC	Q15149	2061.032	LLEAQAcTGGIIDPSTGER	C7	5.446	-
Podocalyxin	PODXL	O00592	947.577	LISLIcR	C6	0.932	-
Poly(rC)-binding protein 1	PCBP1	Q15365	1361.653	INISEGNcPER	C8	1.308	1.235
Poly(rC)-binding protein 1	PCBP1	Q15365	1510.832	LVVPATQcGSLIGK	C8	1.306	-
Poly(rC)-binding protein 1	PCBP1	Q15365	1515.864	LVVPATQcGSLIGK	C8	1.506	1.840
Poly(rC)-binding protein 1	PCBP1	Q15365	1745.952	AITIAGVPQSVTEcVK	C14	2.029	-
Poly(rC)-binding protein 1	PCBP1	Q15365	2548.245	VMTIPYQPMPASSPVIcAGGQDR	C17	1.840	1.298
Polyadenylate-binding protein-interacting protein 1	PAIP1	Q9H074	1462.738	IENVVLDANcSR	C10	0.836	-
Pregnancy zone protein	PZP	P20742	1559.767	DLFHcVSFTLPR	C5	-	-
Prelamin-A/C	LMNA	P02545	1436.674	AQNTWGcGNSLR	C7	1.593	-
Pre-mRNA-processing factor 19	PRPF19	Q9UMS4	1687.899	IWSVPNAScVQVVR	C9	1.421	2.660
Pre-mRNA-splicing factor ATP-dependent RNA helicase DHX15	DHX15	O43143	961.495	GVAcTQPR	C4	0.949	-
Prenylcysteine oxidase 1	PCYOX1	Q9UHG3	1178.601	cPSIILHDR	C1	-	-
Probable ATP-dependent RNA helicase DDX5	DDX5	P17844	1441.791	GDGPIcLVLAPTR	C6	1.388	-
Probable cytosolic iron-sulfur protein assembly protein CIAO1	CIAO1	O76071	991.416	LAScSDDR	C4	-	1.243
Procollagen-lysine,2-oxoglutarate 5-dioxygenase 3	PLOD3	O60568	1164.536	YDcVISSPR	C3	-	1.304
Profilin-1	PFN1	P07737	1239.567	cYEMASHLR	C1	1.450	1.243
Prolow-density lipoprotein receptor-related protein 1	LRP1	Q07954	971.514	VcLWIGR	C2	1.189	1.588

Prolow-density lipoprotein receptor-related protein 1	LRP1	Q07954	1096.448	cEYDGSHR	C1	1.275	1.527
Prolow-density lipoprotein receptor-related protein 1	LRP1	Q07954	1124.613	LDGLcIPLR	C5	1.562	-
Prolow-density lipoprotein receptor-related protein 1	LRP1	Q07954	1163.519	cTAYFEGSR	C1	5.318	-
Prolow-density lipoprotein receptor-related protein 1	LRP1	Q07954	1206.622	ILQDDLTcR	C8	1.342	-
Prolyl 3-hydroxylase 1	P3H1	Q32P28	1418.712	TVTAEVQPQcGR	C10	2.580	1.958
Prolyl 3-hydroxylase 1	P3H1	Q32P28	1943.970	LDTPLYFSYSHLVcR	C14	-	2.789
Prolyl 3-hydroxylase 3	P3H3	Q8IVL6	1894.909	LEEALQGSLAQMEScR	C15	2.143	-
Prostaglandin E synthase 3	PTGES3	Q15185	1518.732	LTFScLGGSDNFK	C5	0.875	-
Proteasome subunit alpha type-5	PSMA5	P28066	2236.035	GPQLFHMDPSGTFVQcDAR	C16	3.524	-
Proteasome subunit alpha type-6	PSMA6	P60900	1928.892	ITENIGcVMTGMTADSR	C7	2.009	-
Protein canopy homolog 2	CNPY2	Q9Y2B0	1848.862	TDLcDHALHISHDEL	C4	10.957	-
Protein CYR61	CYR61	O00622	1172.500	VTNDNPEcR	C8	1.348	0.695
Protein deglycase DJ-1	PARK7	Q99497	934.446	DPVQcSR	C5	1.249	1.867
Protein deglycase DJ-1	PARK7	Q99497	1604.795	DVVIcPDASLEDAK	C5	1.459	-
Protein disulfide-isomerase A5	PDIA5	Q14554	1405.681	NQDLcQQEAVK	C5	2.931	-
Protein flightless-1 homolog	FLII	Q13045	1520.826	VPEcLYTLPSLR	C4	1.324	-
Protein kinase C delta type	PRKCD	Q05655	1010.473	cTGTAANSR	C1	1.858	-
Protein KRBA1	KRBA1	A5PL33	961.404	AEAcESAR	C4	-	1.478
Protein PML	PML	P29590	1284.666	TPTLTSIYcR	C9	1.434	-
Protein PML	PML	P29590	1333.695	QIVDAQAVcTR	C9	-	-
Protein S100-A11	S100A11	P31949	1375.730	cIESLIAVFQK	C1	1.147	1.806
Protein SEC13 homolog	SEC13	P55735	982.467	LATcSSDR	C4	1.828	1.650
Protein SEC13 homolog	SEC13	P55735	2086.963	VFIWTcDDASSNTWSPK	C6	-	-
Protein SEC13 homolog	SEC13	P55735	2462.208	DVAWAPSIGLPTSTIAScSQDGR	C18	1.122	-
Protein transport protein Sec23A	SEC23A	Q15436	1515.765	AVLNPLcQVDYR	C7	1.495	-
Protein transport protein Sec31A	SEC31A	O94979	1280.620	cLSSATDPQTK	C1	-	-
Protein-glutamine gamma-glutamyltransferase 2	TGM2	P21980	1192.517	DHHTADLcR	C8	0.290	0.849
Protein-glutamine gamma-glutamyltransferase 2	TGM2	P21980	2000.959	VVSGMVNcNDDQGVLLGR	C8	0.285	0.758
Putative annexin A2-like protein	ANXA2P2	A6NMY6	1164.527	ALLYLcGGDD	C6	1.443	-
Putative annexin A2-like protein	ANXA2P2	A6NMY6	1845.892	GLGTDEDSLIEIIcSR	C14	1.252	1.213
Putative heat shock 70 kDa protein 7	HSPA7	P48741	1383.621	FEELcSDLFR	C5	2.165	-
Putative nucleoside diphosphate kinase	NME2P1	O60361	1124.557	GDFcIQVGR	C4	1.252	1.272
Putative Ras-related protein Rab-1C	RAB1C	Q92928	834.492	ScLLLR	C2	1.322	1.143
Pyrroline-5-carboxylate reductase 1, mitochondrial	PYCR1	P32322	1513.793	SLLINAVEAScIR	C11	0.391	-
Pyrroline-5-carboxylate reductase 1, mitochondrial	PYCR1	P32322	1518.838	SLLINAVEAScIR	C11	-	-
Pyruvate kinase PKLR	PKLR	P30613	2611.216	AETSDVANAVLDGADcIMLSGETAK	C16	-	-
Pyruvate kinase PKM	PKM	P14618	1432.765	NTGIIcTIGPASR	C6	-	-
Pyruvate kinase PKM	PKM	P14618	1536.708	cDENILWLDYK	C1	1.692	1.837
Pyruvate kinase PKM	PKM	P14618	1950.029	AGKPVIcATQMLESMIK	C7	1.238	1.180
Pyruvate kinase PKM	PKM	P14618	2567.203	AEGSDVANAVLDGADcIMLSGETAK	C16	1.304	1.042
Rab GDP dissociation inhibitor alpha	GDI1	P31150	1906.854	TDDYLDQPcLETVNR	C9	1.038	-

Rab GDP dissociation inhibitor beta	GDI2	P50395	1252.750	VIcILSHPIK	C3	1.339	1.228
Rab GDP dissociation inhibitor beta	GDI2	P50395	1975.877	TDDYLDQPcYETINR	C9	1.181	1.034
Ras-related C3 botulinum toxin substrate 2	RAC2	P15153	1133.604	cVVVGDGAVGK	C1	0.688	-
Ras-related C3 botulinum toxin substrate 2	RAC2	P15153	1313.656	YLEcSALTQR	C4	1.098	1.463
Ras-related protein Rap-1b-like protein	RAP1B	A6NIZ1	1009.432	cDLEDER	C1	1.565	-
Ras-related protein Rap-1b-like protein	RAP1B	A6NIZ1	1627.763	QWNNcAFLESSAK	C5	0.757	1.574
Ras-related protein R-Ras	RRAS	P10301	1155.587	IcSVDGIPAR	C2	0.352	-
Receptor expression-enhancing protein 5	REEP5	Q00765	1138.565	NcMTDLLAK	C2	1.574	1.828
Receptor of activated protein C kinase 1	RACK1	P63244	1434.710	YWLcAATGPSIK	C4	1.470	-
Receptor of activated protein C kinase 1	RACK1	P63244	1980.953	FSPNSSNPIIVScGWDK	C13	1.824	1.031
Receptor of activated protein C kinase 1	RACK1	P63244	2054.928	YTVQDESHSEWVScVR	C14	-	2.073
Reticulon-4	RTN4	Q9NQC3	1636.825	YSNSALGHVNcTIK	C11	0.725	0.648
Reversion-inducing cysteine-rich protein with Kazal motifs	RECK	O95980	1724.667	ScNAQSDQGAMNDMK	C2	0.543	-
RGM domain family member B	RGMB	Q6NW40	994.443	AYAGcTQR	C5	0.721	1.032
Rho-related GTP-binding protein RhoC	RHOC	P08134	1153.671	TcLLIVFSK	C2	-	-
Rho-related GTP-binding protein RhoC	RHOC	P08134	1413.668	ISAFGYLEcSAK	C9	0.819	1.458
Rho-related GTP-binding protein RhoC	RHOC	P08134	1680.931	HFcPNVPIILVGNK	C3	-	-
Ribonuclease inhibitor	RNH1	P13489	1030.525	DLcGIVASK	C3	1.075	0.844
Ribonuclease inhibitor	RNH1	P13489	1222.579	LDDcGLTEAR	C4	1.027	1.222
Ribonuclease inhibitor	RNH1	P13489	1228.588	DSPcQLEALK	C4	1.088	-
Ribonuclease inhibitor	RNH1	P13489	1543.697	LEScGVTSDNcR	C4; C11	-	-
Ribonuclease inhibitor	RNH1	P13489	1581.807	ELcQGLGQPGSVLR	C3	1.100	1.019
Ribonuclease inhibitor	RNH1	P13489	1921.037	WAELLPLLQQcQVVR	C11	1.086	1.745
Ribonuclease inhibitor	RNH1	P13489	2082.055	LGDVGMAELcPGLLHPSSR	C10	1.125	1.224
Ribonuclease inhibitor	RNH1	P13489	2488.283	ELDLSNNcLGDAGILQLVESVR	C8	-	1.388
Ribose-phosphate pyrophosphokinase 1	PRPS1	P60891	1362.674	NcTIVSPDAGGAK	C2	1.256	0.724
Ribose-phosphate pyrophosphokinase 1	PRPS1	P60891	1466.790	VTAVIPcFPYAR	C7	0.840	0.515
Ribose-phosphate pyrophosphokinase 1	PRPS1	P60891	1822.829	FSNQETcVEIGESVR	C7	0.778	0.292
Ribosome-binding protein 1	RRBP1	Q9P2E9	1685.785	LTAEFEEAQTSAcR	C13	1.509	0.960
RNA 3'-terminal phosphate cyclase	RTCA	O00442	1572.922	VSTALScLLGLPLR	C7	1.492	-
RNA polymerase II subunit A C-terminal domain phosphatase SSU72	SSU72	Q9NP77	1306.660	VAVVcSSNQNR	C5	-	2.004
Sarcoplasmic/endoplasmic reticulum calcium ATPase 2	ATP2A2	P16615	1453.767	cLALATHDNPLR	C1	-	-
Sarcoplasmic/endoplasmic reticulum calcium ATPase 2	ATP2A2	P16615	1557.746	TGTLTTNQMoSVcR	C12	-	1.196
Secernin-1	SCRN1	Q12765	1889.851	SSPcIHYFTGTPDPSR	C4	1.154	1.165
Semaphorin-7A	SEMA7A	O75326	1321.659	SEGLLAcGTNAR	C7	2.813	-
Septin-2	Sept2	Q15019	1823.903	LTVVDTPGYGDAINcR	C15	1.585	1.895
Septin-7	Sept7	Q16181	1634.736	ADTLTPEEcQQFK	C9	-	0.934
Serine/arginine-rich splicing factor 1	SRSF1	Q07955	1490.666	EAGDVcYADVYR	C6	2.079	-
Serine/threonine-protein phosphatase 2A 65 kDa regulatory subunit A alpha isoform	PPP2R1A	P30153	1380.631	NLcSDDTPMVR	C3	1.500	1.857

Serine/threonine-protein phosphatase 2A 65 kDa regulatory subunit A alpha isoform	PPP2R1A	P30153	1617.879	ENVIMSQILPcIK	C11	0.948	1.789
Serine/threonine-protein phosphatase 2A catalytic subunit beta isoform	PPP2CB	P62714	1751.839	cGNQAAIMELDDTLK	C1	1.614	-
Serine/threonine-protein phosphatase 2A catalytic subunit beta isoform	PPP2CB	P62714	1776.882	NVVTIFSAPNYcYR	C12	1.113	1.402
Serine/threonine-protein phosphatase 2A catalytic subunit beta isoform	PPP2CB	P62714	1988.891	AHQLVMEGYNWcHDR	C12	-	-
Serine/threonine-protein phosphatase PP1-gamma catalytic subunit	PPP1CC	P36873	1230.570	GNHEcASINR	C5	1.616	1.755
Serine/threonine-protein phosphatase PP1-gamma catalytic subunit	PPP1CC	P36873	1795.889	IcGDIHGQYYDLLR	C2	1.478	1.827
Serpin B6	SERPINB6	P35237	1110.578	TNGILFcGR	C7	-	-
Serpin B6	SERPINB6	P35237	1186.522	ScDFLSSFR	C2	0.702	0.981
Serum albumin	ALB	P02768	1274.551	ccTESLVNR	C1; C2	-	-
Serum albumin	ALB	P02768	1511.672	YIcENQDSISSK	C3	-	0.137
Signal transducer and activator of transcription 1-alpha/beta	STAT1	P42224	1490.747	NLSFFLTPPcAR	C10	0.442	0.799
Small nuclear ribonucleoprotein Sm D2	SNRPD2	P62316	1304.680	NNTQVLINcR	C9	1.174	1.123
Small nuclear ribonucleoprotein Sm D2	SNRPD2	P62316	1316.652	HcNMVLENVK	C2	-	-
Small nuclear ribonucleoprotein-associated proteins B and B'	SNRPB	P14678	929.452	cILQDGR	C1	-	0.536
S-methyl-5'-thioadenosine phosphorylase	MTAP	Q13126	2073.930	TTMRPQSFYDGSHScAR	C15	-	-
Sorting nexin-12	SNX12	Q9UMY4	1734.841	cLHMFLQEEAIDR	C1	-	-
SPARC	SPARC	P09486	1520.693	TFDSScHFFATK	C6	-	-
Spectrin alpha chain, non-erythrocytic 1	SPTAN1	Q13813	1137.527	GAcAGSEDAVK	C3	1.208	-
Spectrin alpha chain, non-erythrocytic 1	SPTAN1	Q13813	1548.775	VNDVcTNGQDLIK	C5	0.884	-
Spectrin alpha chain, non-erythrocytic 1	SPTAN1	Q13813	1553.652	DcEQAENWMAAR	C2	0.736	-
Spliceosome RNA helicase DDX39B	DDX39B	Q13838	1379.727	NcPHIVVGTPGR	C2	1.525	-
Src substrate cortactin	CTTN	Q14247	1160.553	HcSQVDSVR	C2	0.765	-
Staphylococcal nuclease domain-containing protein 1	SND1	Q7KZF4	1166.541	LSEcEEQAK	C4	1.888	1.783
Superoxide dismutase [Cu-Zn]	SOD1	P00441	1074.604	LAcGVIGIAQ	C3	1.215	1.312
Surfeit locus protein 4	SURF4	O15260	1604.834	LcLISTFLEDGIR	C2	-	1.703
Synaptic vesicle membrane protein VAT-1 homolog	VAT1	Q99536	1397.674	cLVLTGFGGYDK	C1	-	-
Synaptic vesicle membrane protein VAT-1 homolog	VAT1	Q99536	1411.685	AcGLNFADLMAR	C2	0.985	0.967
Talin-1	TLN1	Q9Y490	978.484	AVAAGNScR	C8	-	-
Talin-1	TLN1	Q9Y490	1191.531	cTQDLGNSTK	C1	1.824	1.950
Talin-1	TLN1	Q9Y490	1479.712	TVTDMLMTIcAR	C10	-	-
Talin-1	TLN1	Q9Y490	1528.819	ASAGPQPLLVQScK	C13	1.235	-
Talin-1	TLN1	Q9Y490	1541.728	AGALQcSPSDAYTK	C6	-	-
Talin-1	TLN1	Q9Y490	2629.249	MVAAATNNLcEAANAAVQGHASQEK	C10	2.276	-
TAR DNA-binding protein 43	TARDBP	Q13148	1064.502	NPVSQcMR	C6	1.080	-
T-complex protein 1 subunit alpha	TCP1	P17987	1304.716	IcDDELILIK	C2	-	-
T-complex protein 1 subunit alpha	TCP1	P17987	1376.717	VLcELADLQDK	C3	-	-
T-complex protein 1 subunit beta	CCT2	P78371	1627.888	SLHDALcVLAQTVK	C7	1.589	-

T-complex protein 1 subunit delta	CCT4	P50991	1251.653	SIHDALcVIR	C7	1.288	1.167
T-complex protein 1 subunit delta	CCT4	P50991	1256.684	SLHDALcVIR	C7	-	1.121
T-complex protein 1 subunit eta	CCT7	Q99832	1206.569	YNFFTGcPK	C7	-	-
T-complex protein 1 subunit eta	CCT7	Q99832	1625.761	cQVFEETQIGG ER	C1	-	-
T-complex protein 1 subunit gamma	CCT3	P49368	919.544	AcTILLR	C2	1.341	-
T-complex protein 1 subunit gamma	CCT3	P49368	1302.589	NLQDAMQVcR	C9	2.024	1.479
T-complex protein 1 subunit gamma	CCT3	P49368	1406.749	TLIQNcGASTIR	C6	1.902	1.814
Testin	TES	Q9UGI8	966.446	cHELSPR	C1	0.424	-
Tetraspanin-6	TSPAN6	O43657	1091.518	LEDcTPQR	C4	-	1.154
Thioredoxin	TXN	P10599	1278.608	cMPTFQFFK	C1	1.486	-
Thioredoxin domain-containing protein 5	TXNDC5	Q8NBS9	1236.594	IAEVDcTAER	C6	1.091	0.975
Thrombospondin-1	THBS1	P07996	1038.469	ScDSLNNR	C2	2.405	1.696
Thrombospondin-1	THBS1	P07996	1096.510	cEGSSVQTR	C1	2.994	1.643
Thrombospondin-1	THBS1	P07996	1212.597	AQLYIDcEK	C7	-	-
Thrombospondin-1	THBS1	P07996	1405.696	LcNNPTPQFGG K	C2	1.296	0.792
Thrombospondin-1	THBS1	P07996	1646.728	DNcQYVYNVD QR	C3	3.936	0.994
Thrombospondin-1	THBS1	P07996	1732.872	RPPLcYHNGV QYR	C5	-	-
Thrombospondin-1	THBS1	P07996	1748.759	cNYLGHYSDP MYR	C1	2.782	1.130
Thrombospondin-1	THBS1	P07996	2147.894	DNcPFHYNPA QYDYDR	C3	2.124	-
Thrombospondin-1	THBS1	P07996	2168.976	cENTDPGYNcL PcPPR	C1; C10 ; C13	-	-
Thy-1 membrane glycoprotein	THY1	P04216	1635.880	VTSLTAcLVDQ SLR	C7	1.322	2.261
Transcription elongation factor A protein 2	TCEA2	Q15560	1343.645	NcTYTQVQTR	C2	-	-
Transforming protein RhoA	RHOA	P61586	1406.652	IGAFGYMEcSA K	C9	0.740	1.062
Transgelin	TAGLN	Q01995	2182.152	LVEWIIVQcGP DVGRPDR	C9	1.500	1.000
Transgelin-2	TAGLN2	P37802	951.455	NMAcVQR	C4	1.193	2.248
Transitional endoplasmic reticulum ATPase	VCP	P55072	1608.831	LGDVISIQPcPD VK	C10	2.007	0.264
Transitional endoplasmic reticulum ATPase	VCP	P55072	1997.009	QAAPcVLFFDE LDSIAK	C5	-	-
Transketolase	TKT	P29401	1719.823	TVPFcSTFAAF FTR	C5	-	1.124
Transketolase-like protein 2	TKTL2	Q9H0I9	2409.162	FIECIIAEQNMo VSVALGcATR	C18	-	-
Translationally-controlled tumor protein	TPT1	P13693	1505.762	EIADGLcLEVE GK	C7	-	1.194
Transmembrane 9 superfamily member 3	TM9SF3	Q9HD45	1362.662	NLSGQPNFPcR	C10	1.002	0.703
Trifunctional enzyme subunit alpha, mitochondrial	HADHA	P40939	1379.689	cLAPMMSEVIR	C1	-	-
TRIO and F-actin-binding protein	TRIOBP	Q9H2D6	1496.669	ScTDVTEYAV QR	C2	0.945	2.380
Triosephosphate isomerase	TPI1	P60174	1210.631	IAVAAQNcYK	C8	-	1.616
Triosephosphate isomerase	TPI1	P60174	1394.700	IIYGGSVTGAT cK	C12	0.836	1.623
Triosephosphate isomerase	TPI1	P60174	1654.769	DcGATWVVLG HSER	C2	0.948	1.545
Triosephosphate isomerase	TPI1	P60174	1881.031	VAHALAEGLG VIAcIGEK	C14	0.900	1.356
Triosephosphate isomerase	TPI1	P60174	2265.130	VPADTEVVcAP PTAYIDFAR	C9	0.899	1.261
Tripeptidyl-peptidase 2	TPP2	P29144	1496.661	AcIDSNEDGDL SK	C2	-	-
Tropomyosin alpha-1 chain	TPM1	P09493	1193.579	cAELEEELK	C1	6.455	-
Tropomyosin alpha-4 chain	TPM4	P67936	1165.548	cGDLEEELK	C1	1.658	-
Tropomyosin alpha-4 chain	TPM4	P67936	2413.176	EENVGLHQTL DQTLNELNcI	C19	-	2.637

Tubulin alpha-1C chain	TUBA1C	Q9BQE3	1671.826	TIQFVDWcPTGFK	C8	1.315	0.963
Tubulin alpha-1C chain	TUBA1C	Q9BQE3	1923.953	AVcMLSNTTAVAEAWAR	C3	1.159	1.309
Tubulin alpha-4A chain	TUBA4A	P68366	1385.604	YMAccLLYR	C4; C5	-	-
Tubulin alpha-4A chain	TUBA4A	P68366	1652.779	SIQFVDWcPTGFK	C8	1.112	1.074
Tubulin alpha-4A chain	TUBA4A	P68366	1932.933	AVcMLSNTTAIAEAWAR	C3	1.141	1.211
Tubulin alpha-4A chain	TUBA4A	P68366	1948.926	AVcMoLSNTTAIAEAWAR	C3	0.982	1.993
Tubulin alpha-4A chain	TUBA4A	P68366	2823.354	AYHEQLSVAEITNAcFEPANQMVK	C15	1.210	1.764
Tubulin beta-3 chain	TUBB3	Q13509	1099.599	VAVcDIPPR	C4	-	-
Tubulin beta-4A chain	TUBB4A	P04350	1101.577	TAVcDIPPR	C4	1.167	-
Tubulin beta-4A chain	TUBB4A	P04350	1890.954	EIVHLQAGQcGNQIGAK	C10	-	0.966
Tubulin beta-4A chain	TUBB4A	P04350	2781.398	LTTPTYGDLNHLVSATMSGVTTcLR	C23	1.245	1.116
Tubulin beta-4A chain	TUBB4A	P04350	2797.392	LTTPTYGDLNHLVSATMoSGVTTcLR	C23	1.109	-
Tubulin beta-6 chain	TUBB6	Q9BUF5	2855.335	ALTVPELTQQMFDARNMoMAAcDPR	C21	-	-
Tubulin-folding cofactor B	TBCB	Q99426	2095.968	LDQEDALLGSYPVDDGcR	C17	1.569	0.775
Ubiquitin carboxyl-terminal hydrolase 14	USP14	P54578	1413.611	cTESEEEEVTK	C1	2.099	-
Ubiquitin carboxyl-terminal hydrolase isozyme L1	UCHL1	P09936	968.529	FSAVALcK	C7	2.973	0.469
Ubiquitin carboxyl-terminal hydrolase isozyme L1	UCHL1	P09936	2040.960	NEAIQAAHDAVAQEGQcR	C17	-	-
Ubiquitin-60S ribosomal protein L40	UBA52	P62987	1269.653	cGHTNNLRPK	C1	-	-
Ubiquitin-conjugating enzyme E2 D1	UBE2D1	P51668	2167.098	IYHPNINSNGSIcLDILR	C13	-	0.473
Ubiquitin-conjugating enzyme E2 N	UBE2N	P61088	947.565	IcLDILK	C2	1.411	-
Ubiquitin-like modifier-activating enzyme 1	UBA1	P22314	1091.509	VGEFcHNR	C5	1.164	0.679
Ubiquitin-like modifier-activating enzyme 1	UBA1	P22314	1518.729	DNPGVVTcLDEAR	C8	2.162	-
Uroporphyrinogen decarboxylase	UROD	P06132	1270.554	AAQDFFSTcR	C9	1.440	0.477
Vacuolar protein sorting-associated protein 35	VPS35	Q96QK1	1022.535	TQcALAASK	C3	-	-
Vacuolar protein sorting-associated protein 35	VPS35	Q96QK1	1140.588	NYLLQcTR	C6	-	1.047
Valine--tRNA ligase	VARS	P26640	1241.676	IcLQPPPTSR	C2	-	-
Versican core protein	VCAN	P13611	1155.594	AQcGGGLLGVR	C3	-	0.824
Vesicular integral-membrane protein VIP36	LMAN2	Q12907	1120.584	NcIDITGVR	C2	1.871	-
Vesicular integral-membrane protein VIP36	LMAN2	Q12907	1499.700	WTELAGcTADFR	C7	2.085	1.283
Vigilin	HDLBP	Q00341	976.420	DcDPGSPR	C2	2.657	-
Vigilin	HDLBP	Q00341	1119.625	cDIIIISGR	C1	-	-
Vigilin	HDLBP	Q00341	1135.603	IcLEIMQR	C2	1.798	-
Vigilin	HDLBP	Q00341	1275.726	RcDIIIISGR	C2	-	2.118
Vimentin	VIM	P08670	1563.815	QVQSLTcEVDALK	C7	1.321	1.440
Vinculin	VCL	P18206	1133.555	IAELcDDPK	C5	-	0.230
Vinculin	VCL	P18206	1205.636	TNLLQVcER	C7	-	-
Vinculin	VCL	P18206	1989.026	cDRVDQLTAQLADLAAR	C1	1.506	-
WD repeat-containing protein 1	WDR1	O75083	864.465	VcALGGSK	C2	-	-
WD repeat-containing protein 1	WDR1	O75083	2116.972	LATGSDDNcAAFFEGPPFK	C9	-	-
WD repeat-containing protein 1	WDR1	O75083	2211.963	MTVDESGQLIScSMDDTVR	C12	1.567	1.035

WD repeat-containing protein 1	WDR1	O75083	3470.680	cFSIDNPGYEPE VVAVHPGGDT VAIGGVDGNV R	C1	-	-
WD repeat-containing protein 3	WDR3	Q9UNX4	1420.673	IWGLDFGDcH K	C9	-	-
X-ray repair cross-complementing protein 5	XRCC5	P13010	1159.647	LFQcLLHR	C4	1.185	-
Zinc finger CCCH-type antiviral protein 1	ZC3HAV1	Q7Z2W4	1116.479	cNYSQSER	C1	-	-
Zyxin	ZYX	Q15942	949.468	cHQPLAR	C1	1.472	1.433
Zyxin	ZYX	Q15942	1332.674	QNVAVNELcG R	C9	1.251	1.320

Appendix Table S2. Acylcarnitine profile in blood of Pdss2 $^{kd/kd}$ mice

**Subject ID**	**C0-AC (ng/ml)**	**C2-AC (ng/ml)**	**C3-AC (ng/ml)**	**C04-AC (ng/ml)**	**C5-AC (ng/ml)**	**C6-AC (ng/ml)**
WT	2002.46 ± 173.42	3855.64 ± 408.78	609.39 ± 128.9	279.84 ± 64.74	30.31 ± 4.2	50.24 ± 10.23
MUT	2041.014 ± 172	4541.35 ±645.82	716.1 ± 168.7	659.17 ± 153.1	50.6 ± 12.95	114.68 ± 45.9
**Subject ID**	**C8-AC (ng/ml)**	**C8:1-AC (ng/ml)**	**C10-AC (ng/ml)**	**C10:1-AC (ng/ml)**	**C10:2-AC (ng/ml)**	**C12-AC (ng/ml)**
WT	5.43 ± 0.819	<1.00	4.84 ± 0.86	3.78 ± 0.33	<1.00	4.05 ± 0.36
MUT	6.45 ± 1.94	<1.00	4.37 ± 1.66	3.86 ± 1.58	<1.00	4.42 ± 1.85
**Subject ID**	**C12:1-AC (ng/ml)**	**C14-AC (ng/ml)**	**C14:1-AC (ng/ml)**	**C14:2-AC (ng/ml)**	**C16-AC (ng/ml)**	**C16:1-AC (ng/ml)**
WT	1.76 ± 0.56	13.88 ± 0.88	12.06 ± 1.96	3.53 ± 0.3	19.93 ± 1.38	4.1 ± 0.62
MUT	4.29 ± 3.95	18.9 ± 5.7	18.51 ± 12.16	5.39 ± 3.87	37.2 ± 12.68	9.23 ± 4.63
