# Supplementary material for: Coenzyme Q deficiency causes impairment of the sulfide oxidation pathway
Source: EMBO Mol Med. 2016 Nov 17;9(1):96–111. doi: 10.15252/emmm.201606356 (PMC5210092; doi:10.15252/emmm.201606356)
Supplement: Supplementary file 2 — Expanded View Figures PDF [file EMMM-9-96-s002.pdf]

Expanded View Figures

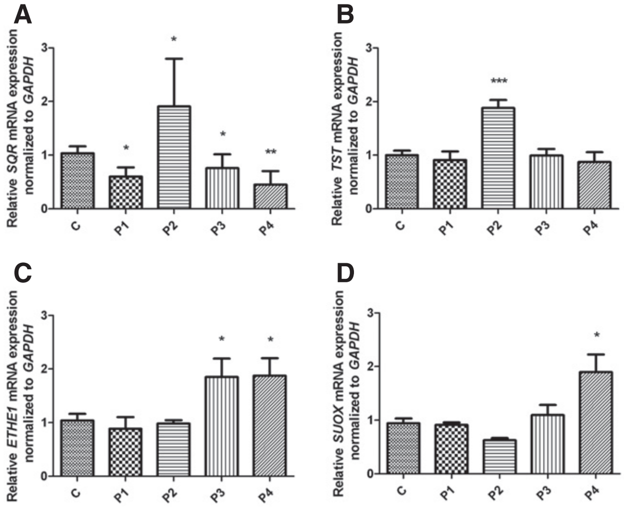

Figure EV1. *SQR*, *TST*, *ETHE1*, and *SUOX* mRNA transcript levels in patient fibroblasts.

A–D Relative levels of (A) *SQR*, (B) *TST*, (C) *ETHE1*, and (D) *SUOX* transcripts normalized to *GAPDH* in control (C, $n = 5$) and patient fibroblasts (P1–P4). Error bars represent SDs of three experiments. Mann–Whitney *U*-test. * indicates a value of $P < 0.05$, ** indicates a value of $P < 0.01$, and *** indicates a value of $P < 0.001$.

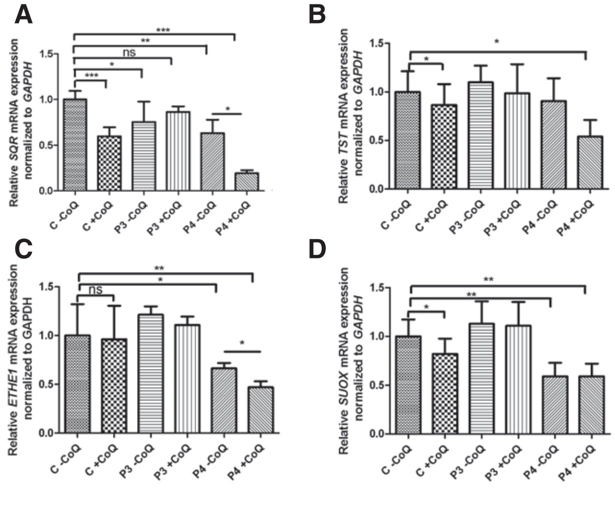

Figure EV2. *SQR*, *TST*, *ETHE1*, and *SUOX* mRNA in fibroblasts after CoQ supplementation.

A–D Relative levels of (A) *SQR*, (B) *TST*, (C) *ETHE1*, and (D) *SUOX* transcripts normalized to *GAPDH* in control (C, $n = 2$) and patient fibroblasts (P3 and P4). Mann–Whitney *U*-test. Error bars represent SDs of five experiments. * indicates a value of $P < 0.05$, ** indicates a value of $P < 0.01$, and *** indicates a value of $P < 0.001$.

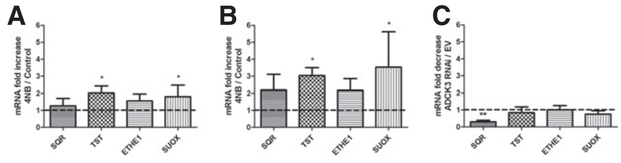

Figure EV3. *SQR*, *TST*, *ETHE1*, and *SUOX* mRNA in 4-NB-treated fibroblasts and ADCK3-depleted HeLa cells.

A Fibroblasts supplemented with 4-NB in DMEM high-glucose medium. − indicates cells treated with DMSO, + indicates cells treated with DMSO + 4-NB.

B Fibroblasts supplemented with 4-NB in galactose medium. mRNA levels are normalized to *GAPDH*.

C *SQR*, *TST*, *ETHE1*, and *SUOX* transcripts normalized to *GAPDH* in ADCK3-depleted clones (ADCK3 RNAi) represented as fold decrease compared with control clones (EV).

Data information: Error bars represent SDs of three different experiments. Paired *t*-test in (A and B). Mann–Whitney *U*-test in (C). * indicates a value of $P < 0.05$. ** indicates a value of $P < 0.01$.

© 2016 The Authors

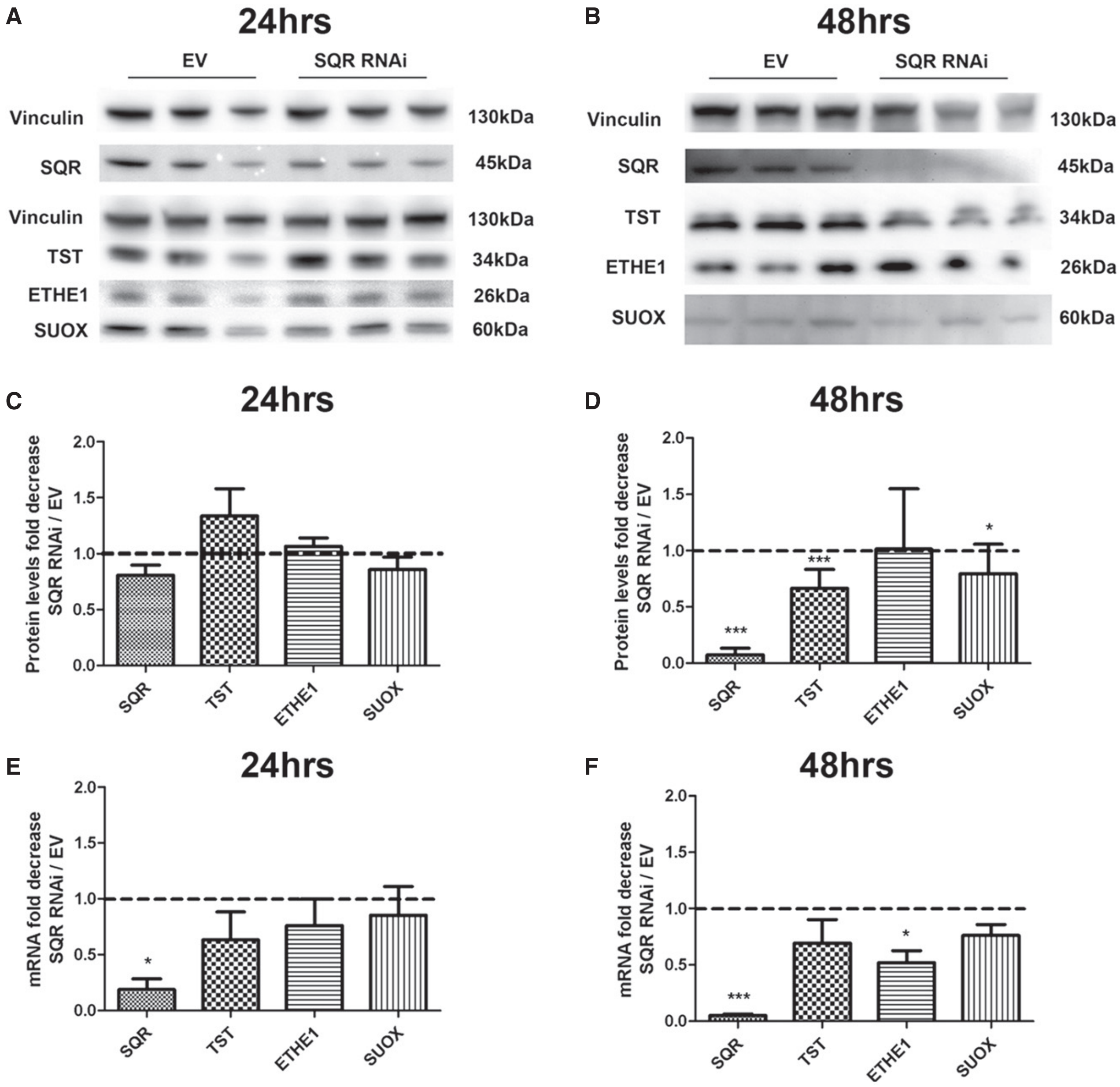

Figure EV4. SQR, TST, ETHE1, and SUOX protein and mRNA levels in SQR-depleted HeLa cells.

A, B Representative Western blots showing the level of SQR, TST, ETHE1, and SUOX proteins in control (EV) and SQR-depleted cells (SQR RNAi) after 24 h (A) and 48 h (B) of treatment.

C, D Proteins are normalized to vinculin and represented as fold decrease compared with controls.

E, F *SQR, TST, ETHE1*, and *SUOX* transcripts normalized to *GAPDH* and represented as fold decrease in *SQR*-depleted compared with control (EV) cell lines.

Data information: Error bars in (C–F) represent SDs of three experiments. Mann–Whitney *U*-test. * indicates a value of $P < 0.05$, and *** indicates a value of $P < 0.001$. Source data are available online for this figure.

 © 2016 The Authors

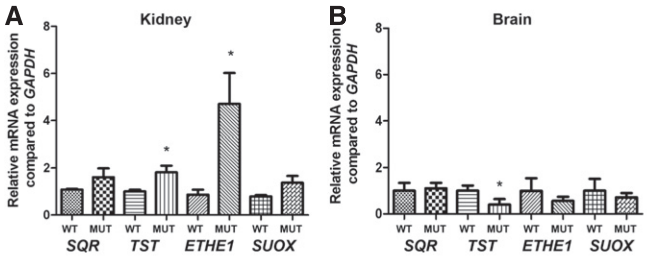

Figure EV5. *SQR*, *TST*, *ETHE1*, and *SUOX* mRNA transcript levels in *Pdss2*$^{kd/kd}$ mice.

A Transcript levels in kidneys of wild-type (WT) and mutant (Mut) mice.
B Transcript levels in brains of wild-type (WT) and mutant (Mut) mice.

Data information: mRNA levels are normalized to *GAPDH*. Five to ten mice were used for each group. Error bars represent SDs of three experiments. Mann–Whitney *U*-test. * indicates a value of $P < 0.05$.
