# Supplementary material for: Coenzyme Q deficiency causes impairment of the sulfide oxidation pathway
Source: EMBO Mol Med. 2016 Nov 17;9(1):96–111. doi: 10.15252/emmm.201606356 (PMC5210092; doi:10.15252/emmm.201606356)

SourceDataForFigureEV4A: Unedited membranes for SQR, SUOX, TST and ETHE1 western blots

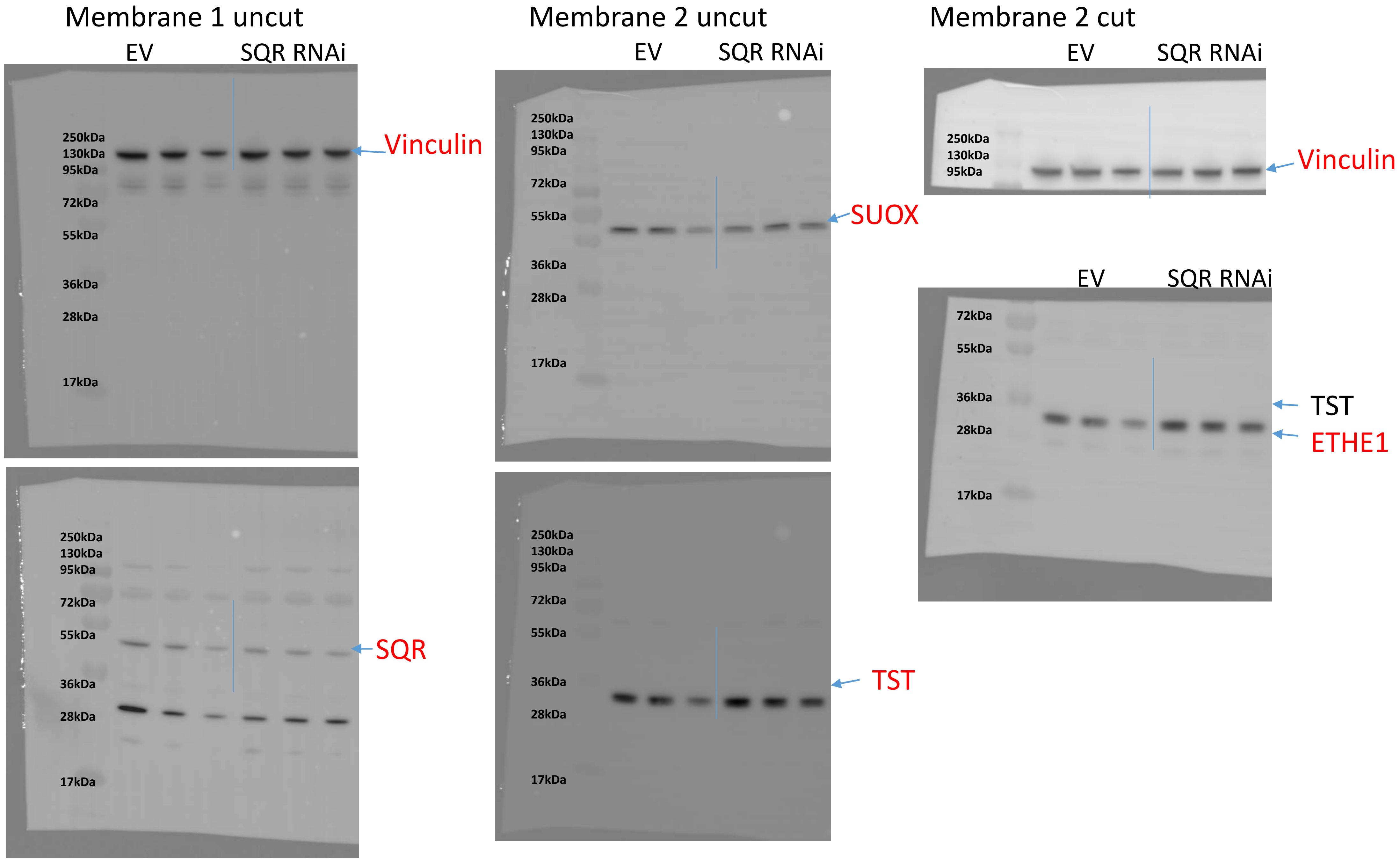

SourceDataForFigureEV4B: Unedited membrane for SQR, SUOX, TST and ETHE1 western blots

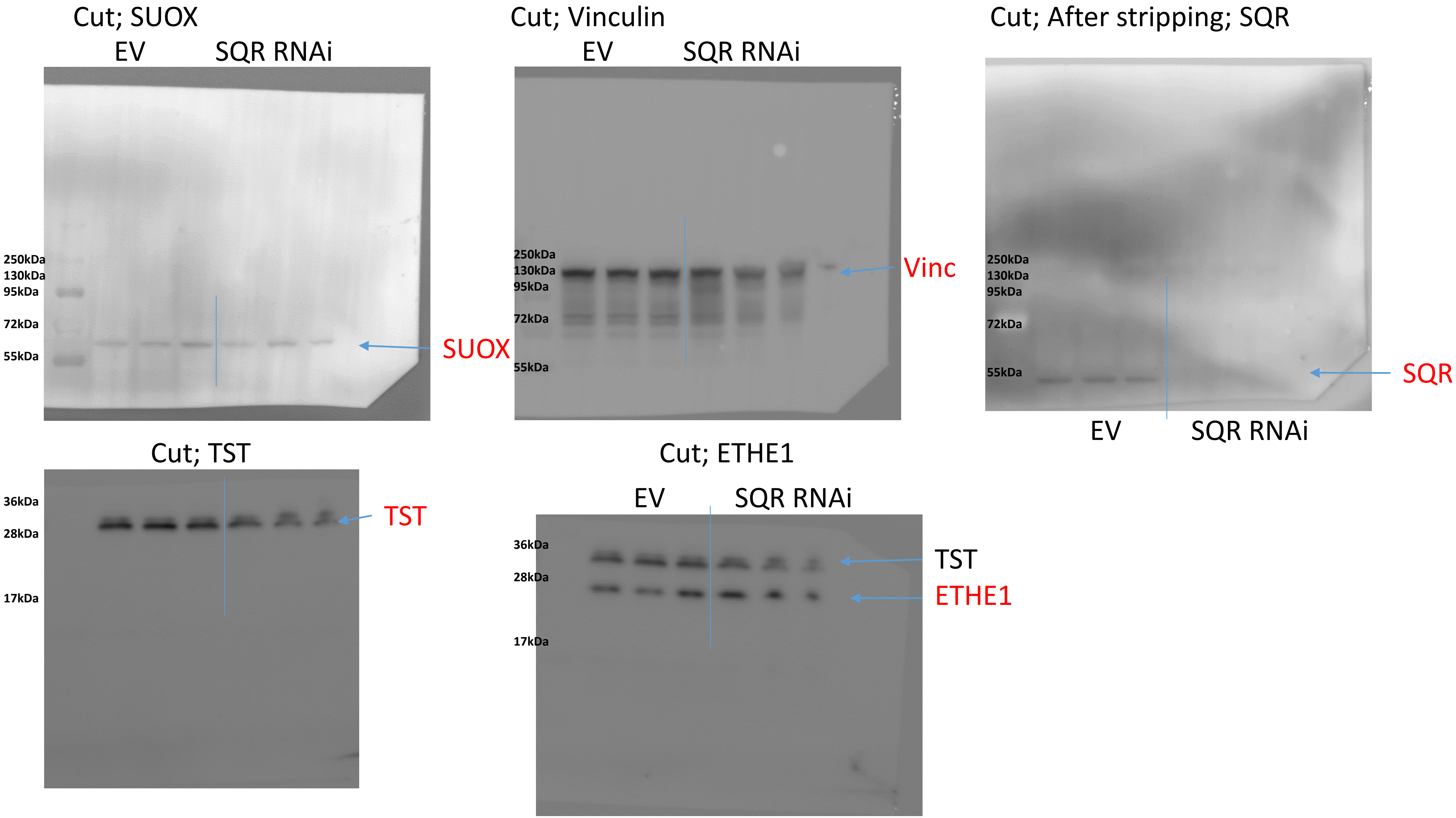

Supplement: Supplementary file 3 — Source Data for Expanded View and Appendix [file EMMM-9-96-s008.zip › EMM_6356_SourceData_EV_Appendix/Source_data_for_Figure_EV4.pdf]
