# Supplementary material for: Coenzyme Q deficiency causes impairment of the sulfide oxidation pathway
Source: EMBO Mol Med. 2016 Nov 17;9(1):96–111. doi: 10.15252/emmm.201606356 (PMC5210092; doi:10.15252/emmm.201606356)

SourceDataForFigure4A: Unedited membrane for SQR and TST western blots

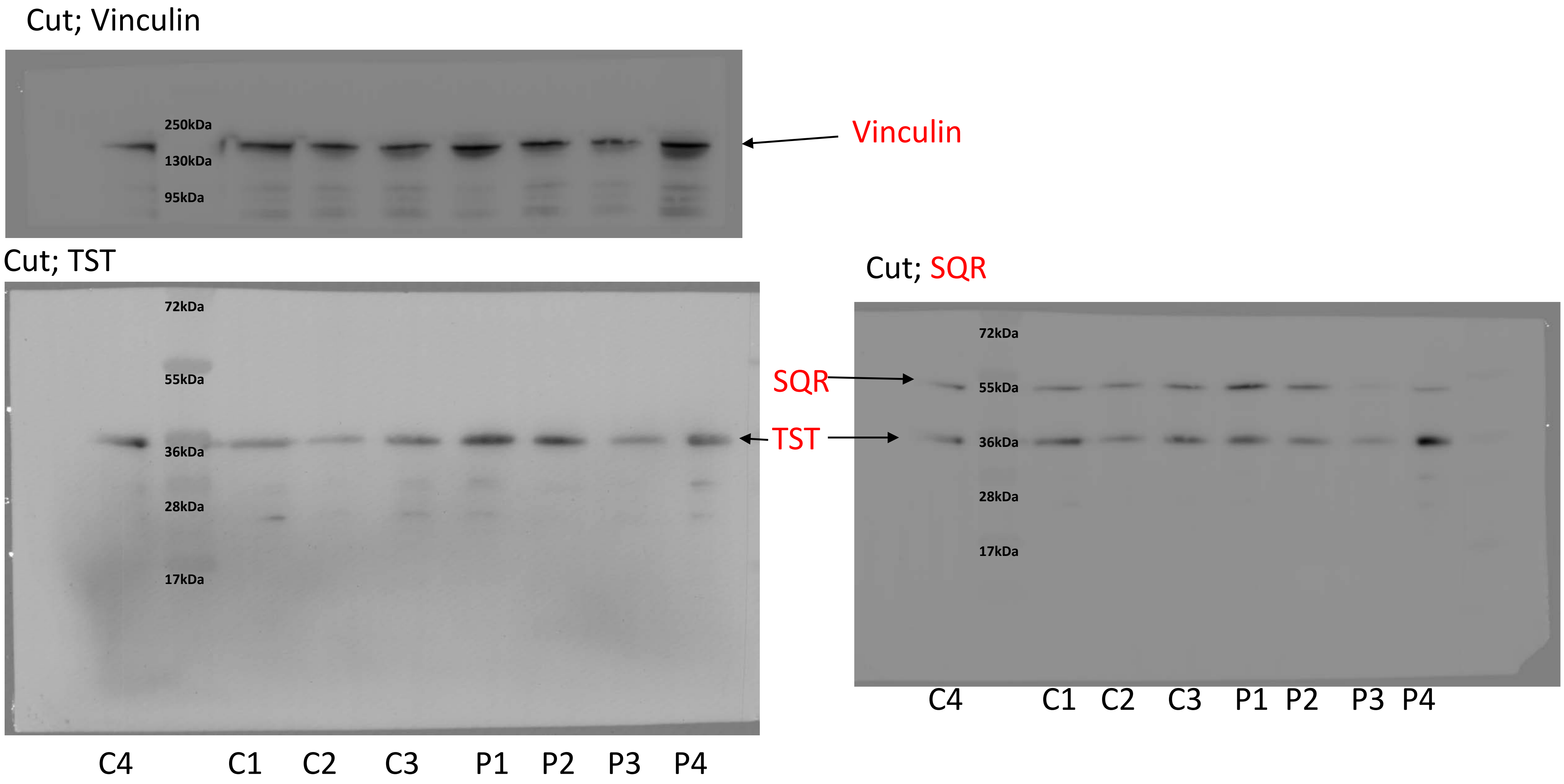

SourceDataForFigure4A: Unedited membrane for SUOX western blots

Uncut Vinculin

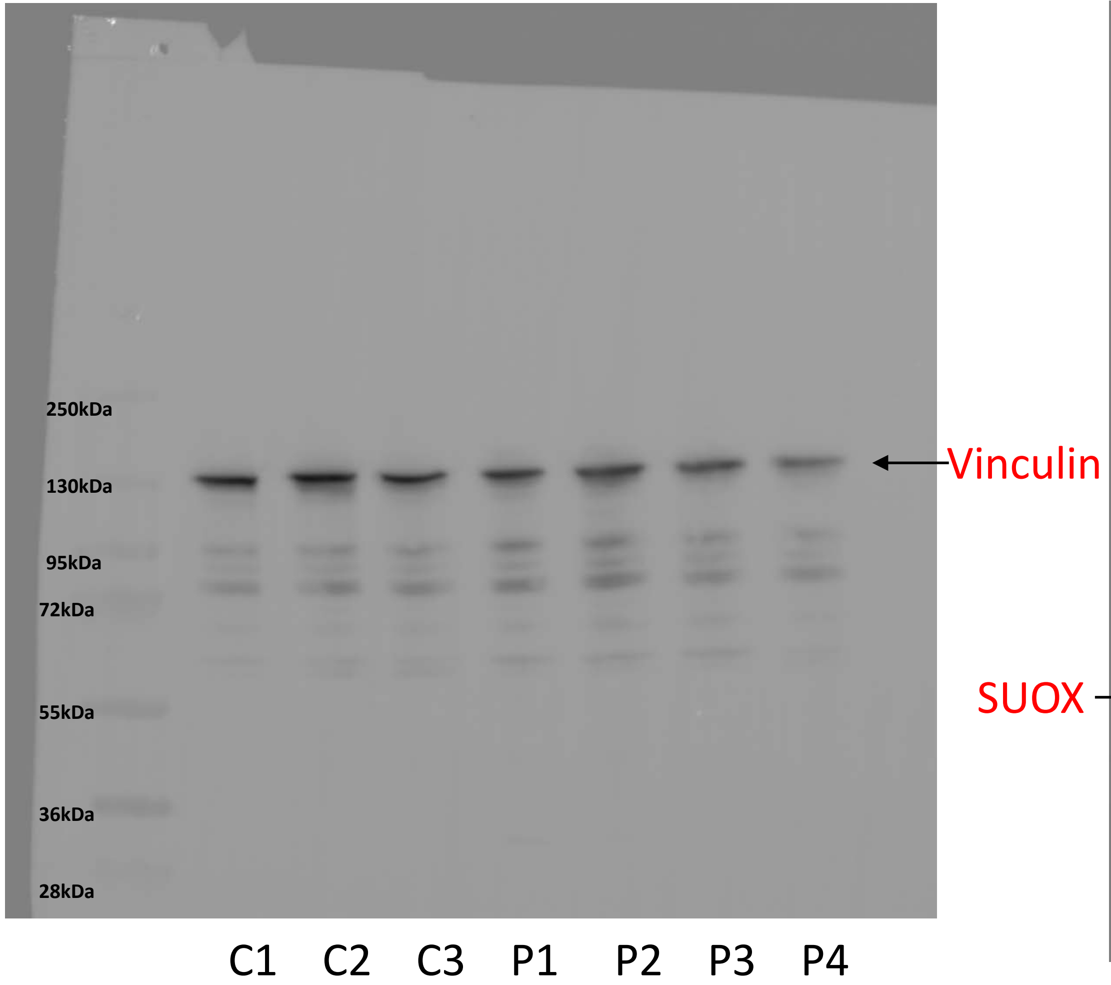

Uncut SUOX

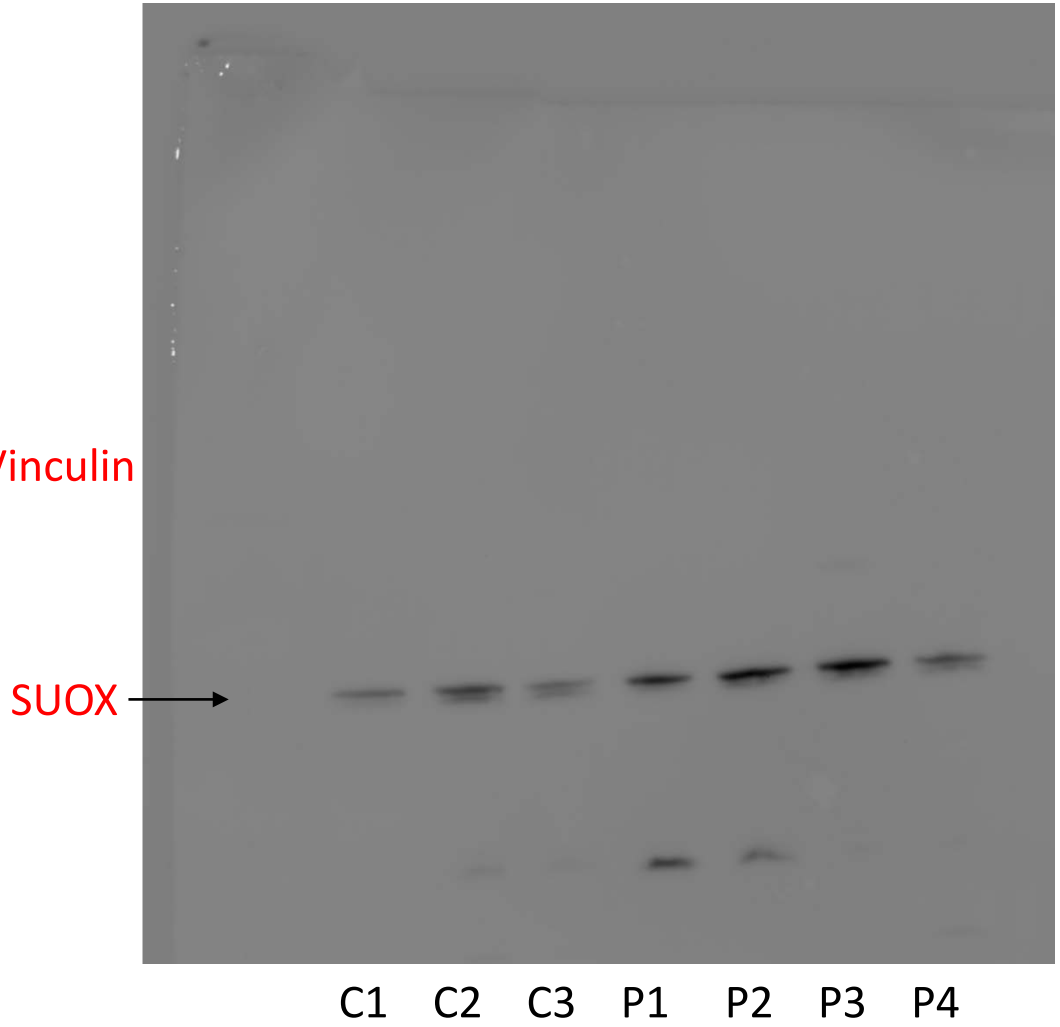

SourceDataForFigure4A: Unedited membrane for ETHE1 western blot

Cut; Vinculin

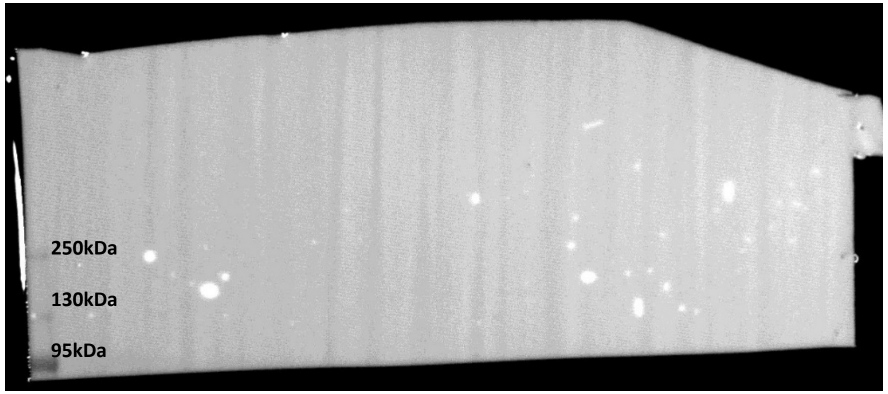

Vinculin

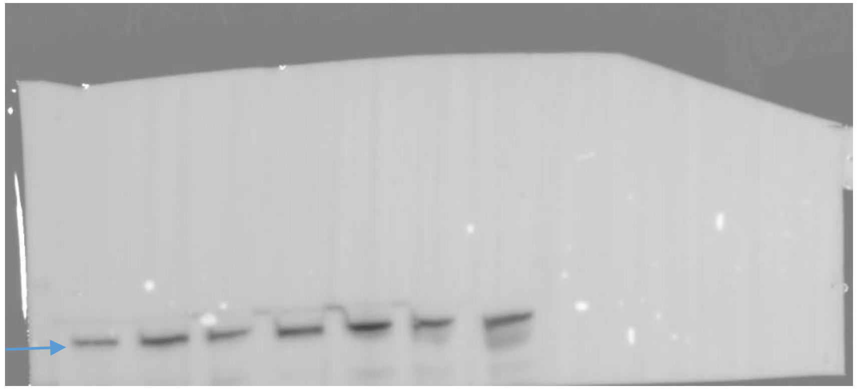

C1 C2 C3 P1 P2 P3 P4

Cut; ETHE1

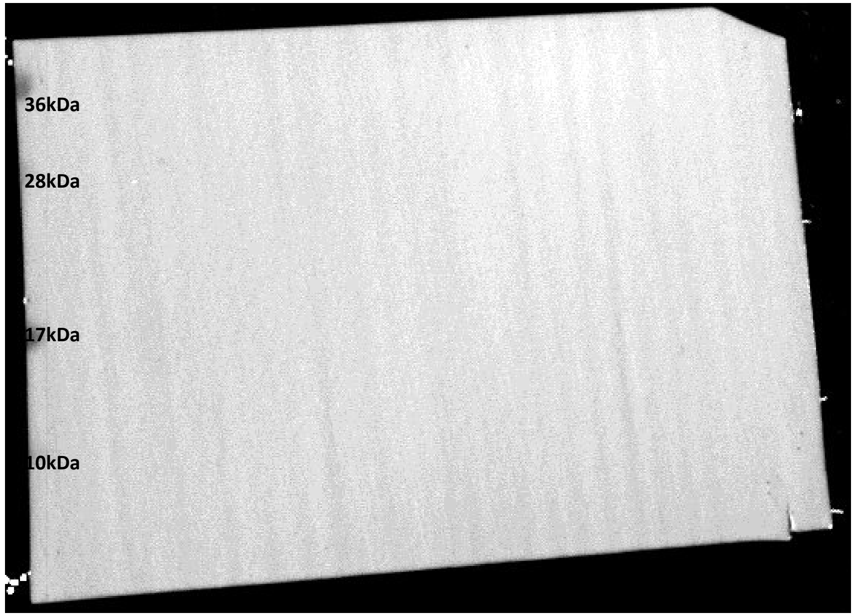

ETHE1

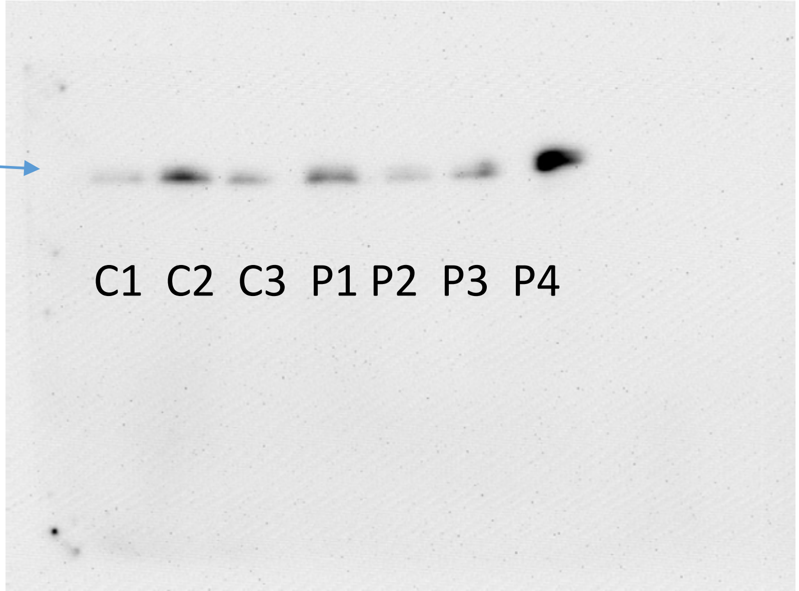

Supplement: Supplementary file 5 — Source Data for Figure 4 [file EMMM-9-96-s003.pdf]
