# Supplementary material for: Coenzyme Q deficiency causes impairment of the sulfide oxidation pathway
Source: EMBO Mol Med. 2016 Nov 17;9(1):96–111. doi: 10.15252/emmm.201606356 (PMC5210092; doi:10.15252/emmm.201606356)

SourceDataForFigure5A: Unedited membrane for SQR and SUOX Western blots

Not exposed; top membrane

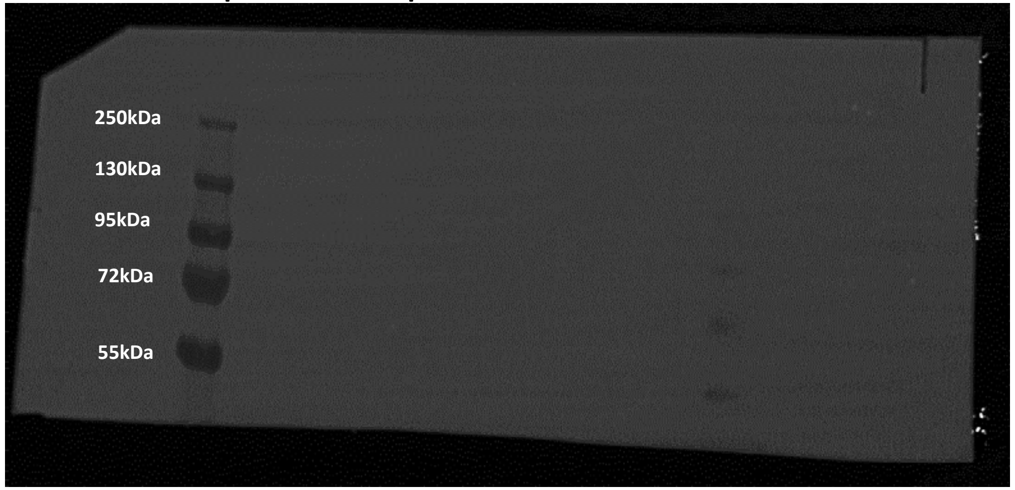

Cut; Vinculin

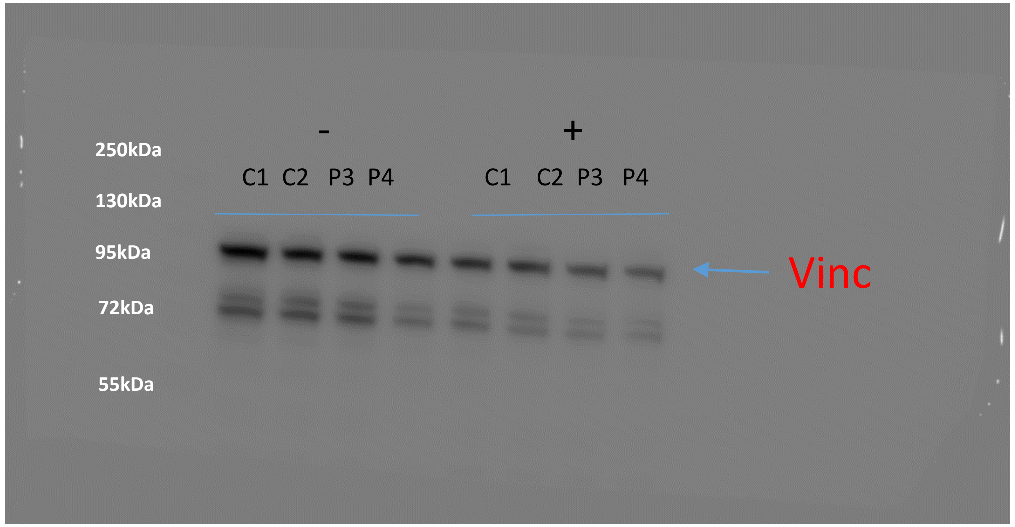

Cut; SQR

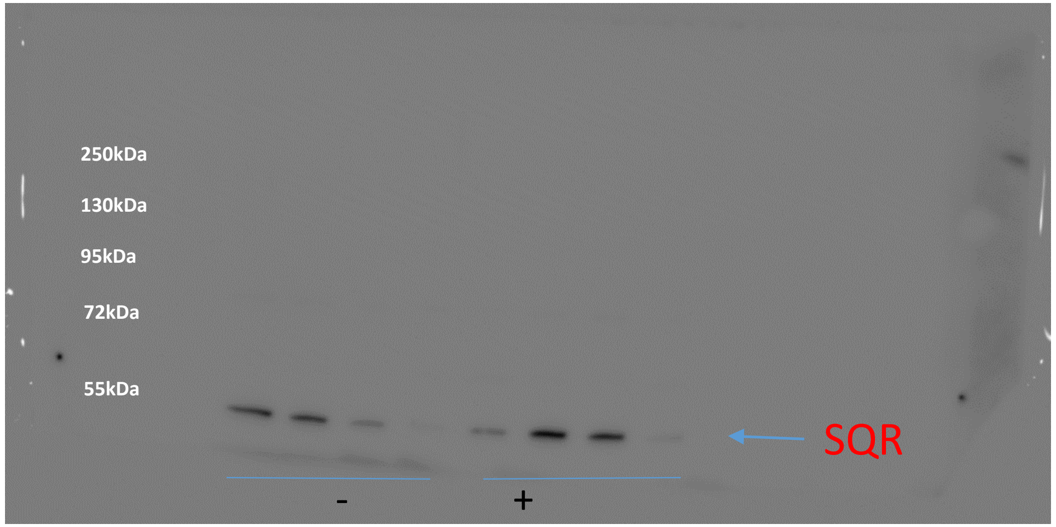

C1 C2 P3 P4 C1 C2 P3 P4

Cut; SUOX

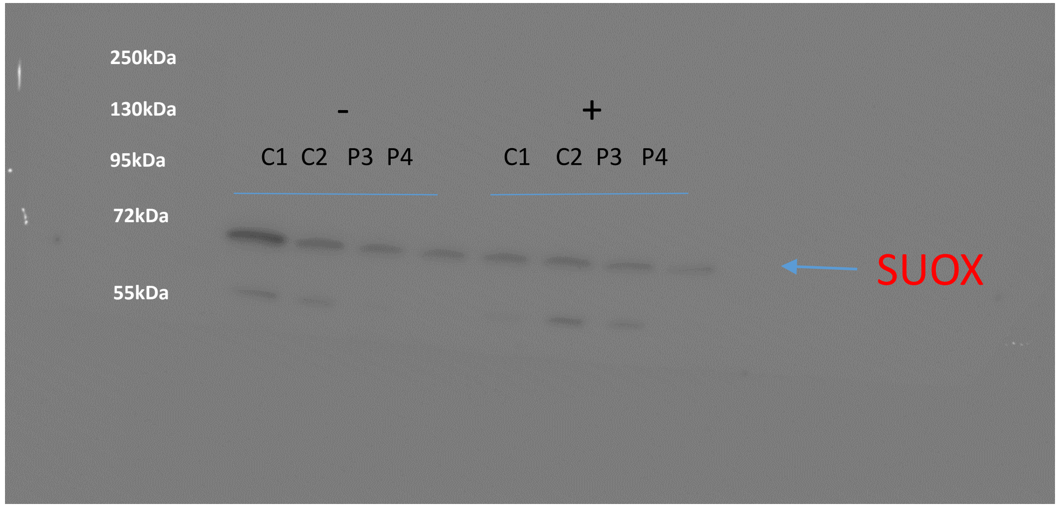

SourceDataForFigure5A: Unedited membrane for TST and ETHE1 Western blots

Not exposed; top membrane

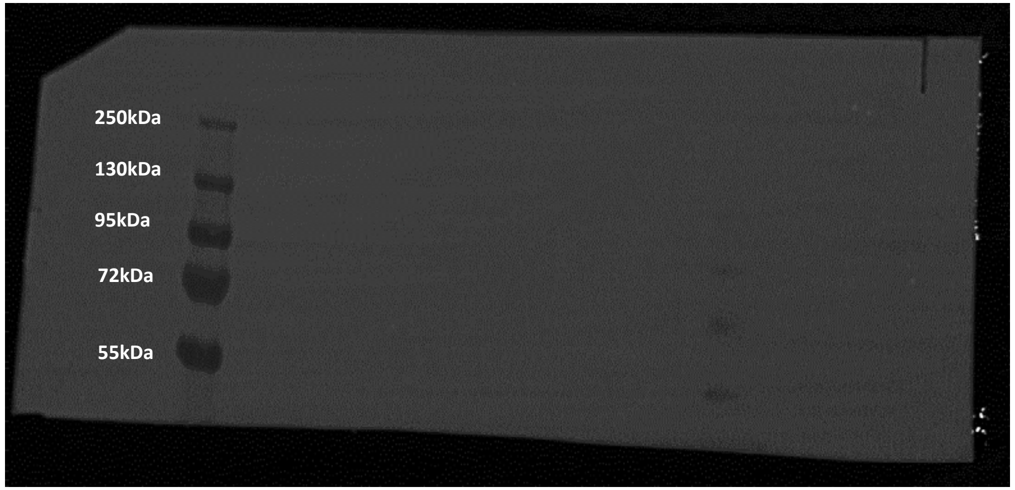

Cut; TST

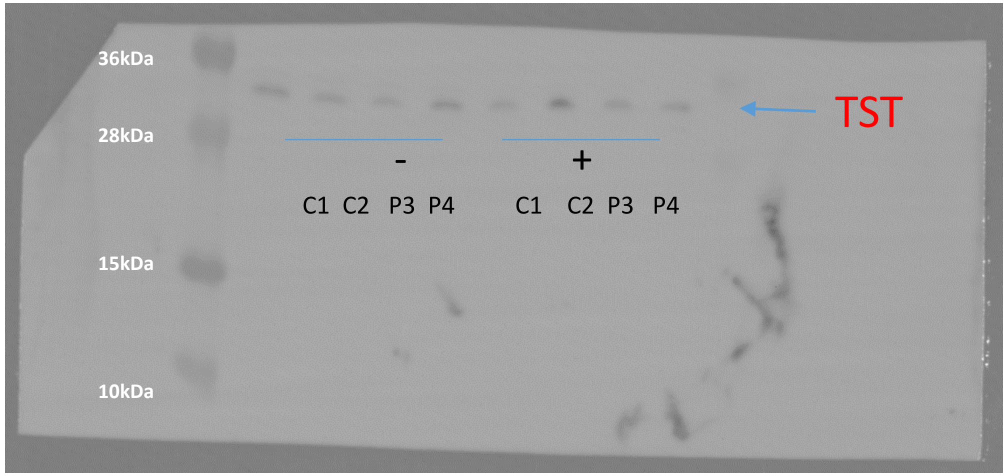

Not exposed; bottom membrane

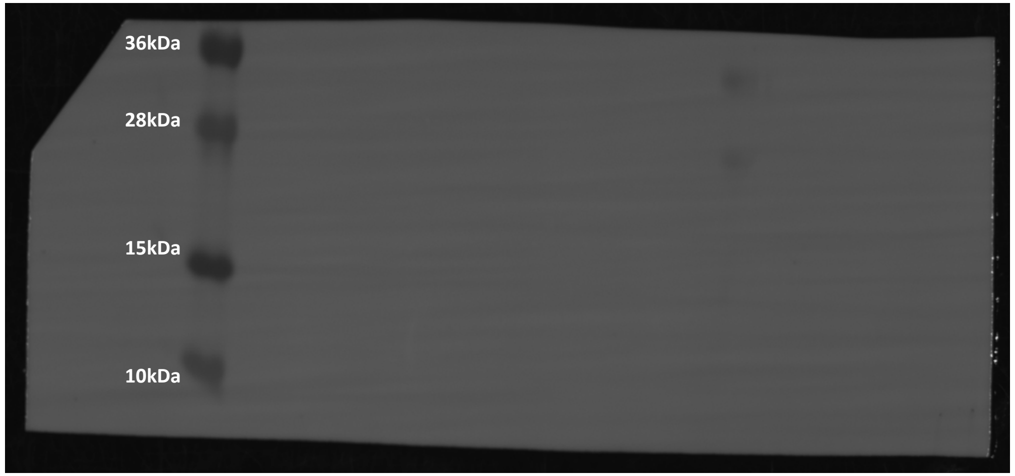

Cut; ETHE1

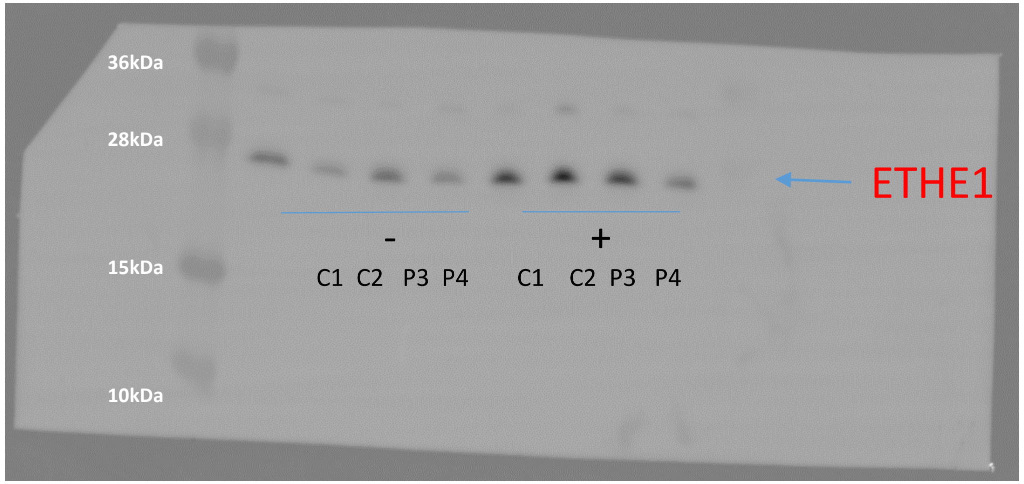

Supplement: Supplementary file 6 — Source Data for Figure 5 [file EMMM-9-96-s004.pdf]
