# Supplementary material for: Coenzyme Q deficiency causes impairment of the sulfide oxidation pathway
Source: EMBO Mol Med. 2016 Nov 17;9(1):96–111. doi: 10.15252/emmm.201606356 (PMC5210092; doi:10.15252/emmm.201606356)

SourceDataForFigure6A: Unedited membranes for SQR, TST, SUOX and ETHE1 western blots

Fig. 6A

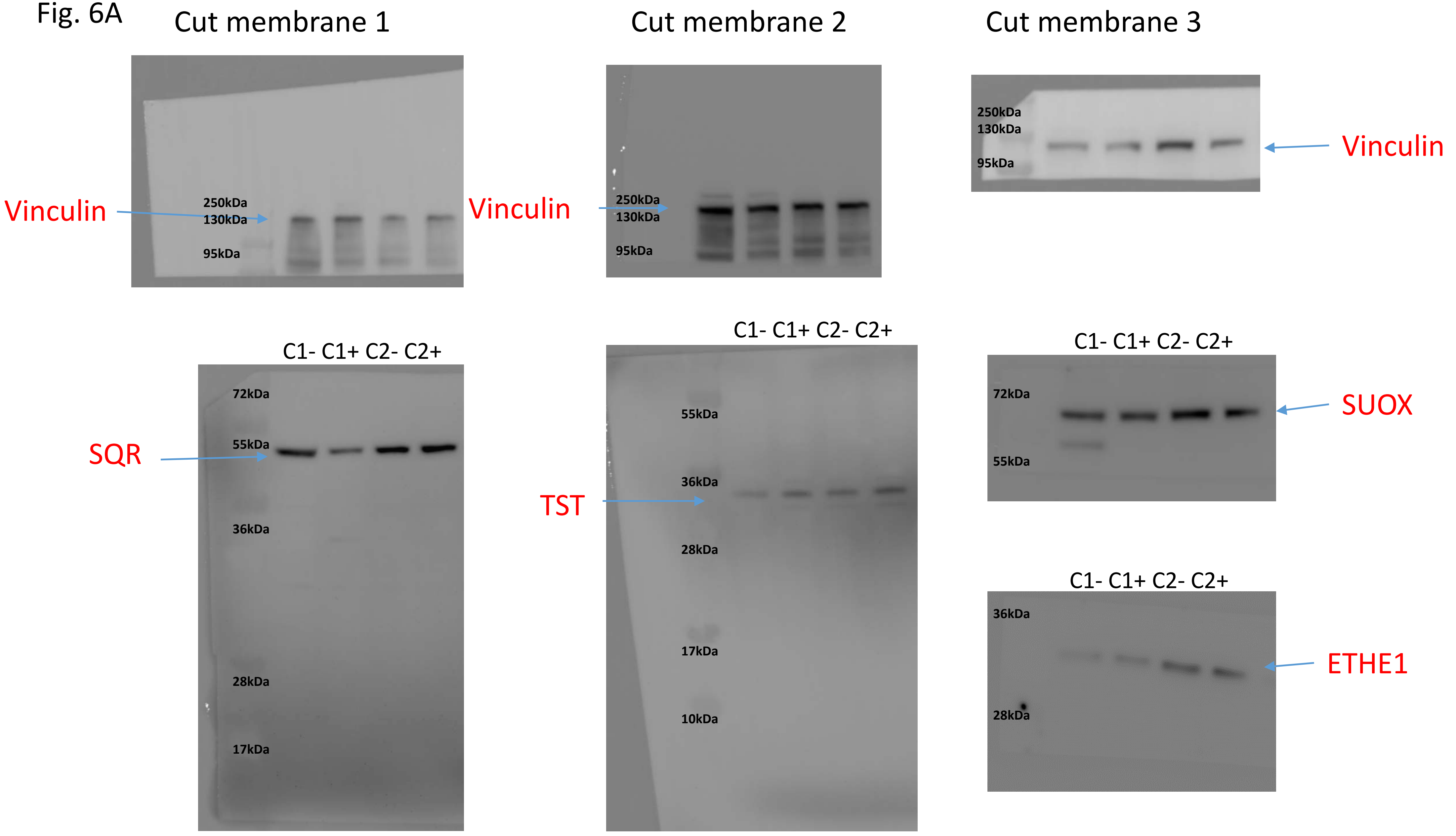

SourceDataForFigure6C: Unedited membrane for ADCK3 and SQR western blots

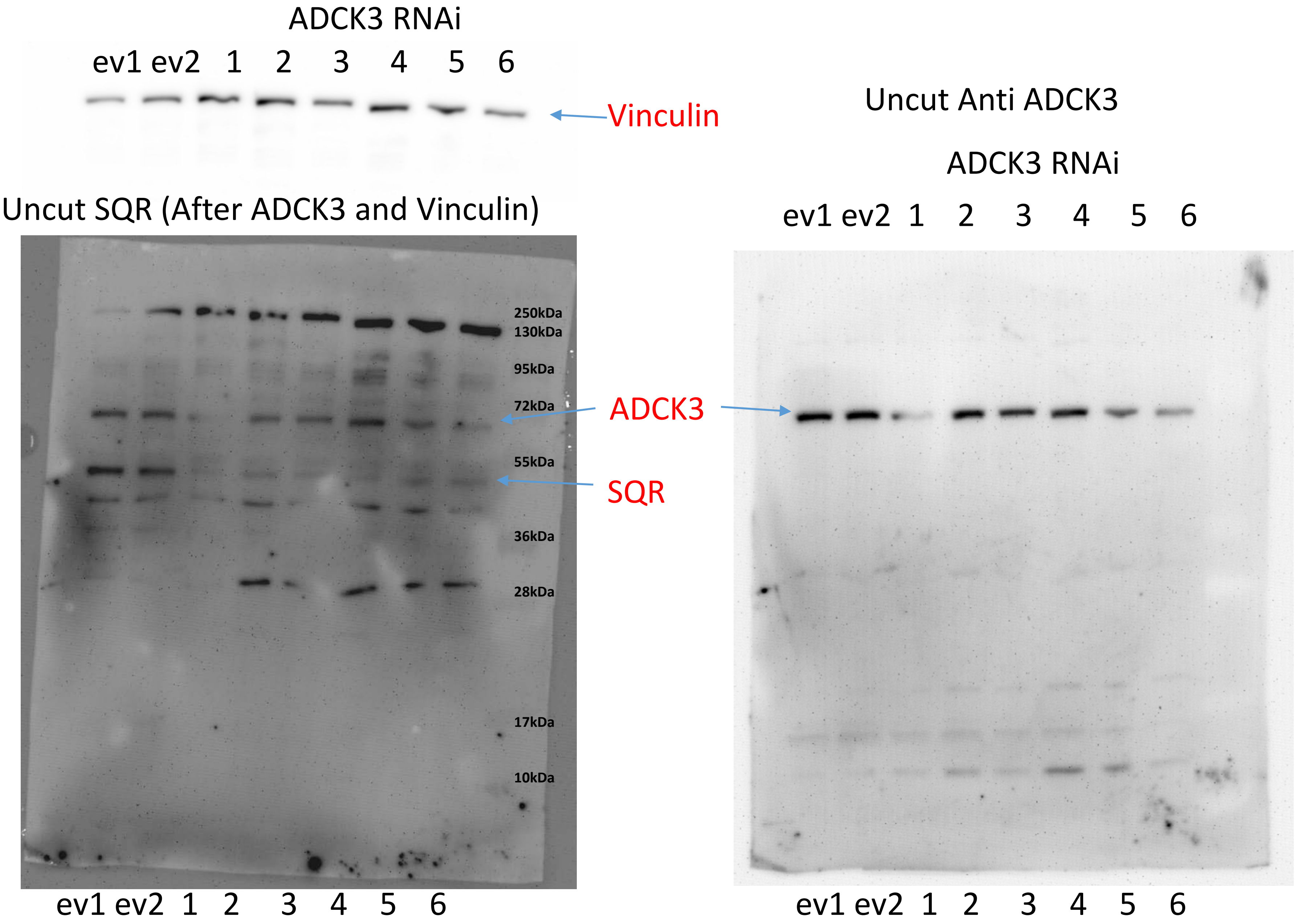

SourceDataForFigure6C: Unedited membrane for SUOX, ETHE1 and TST western blots

Uncut

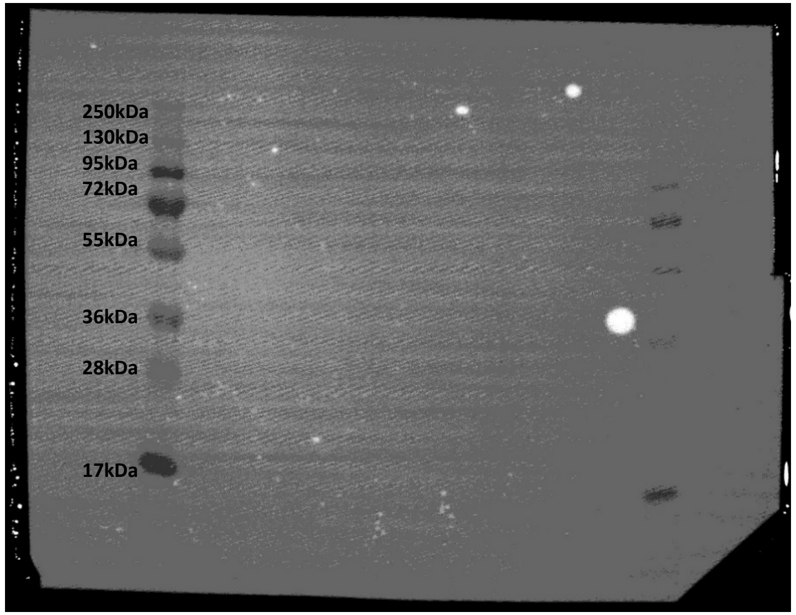

Uncut SUOX

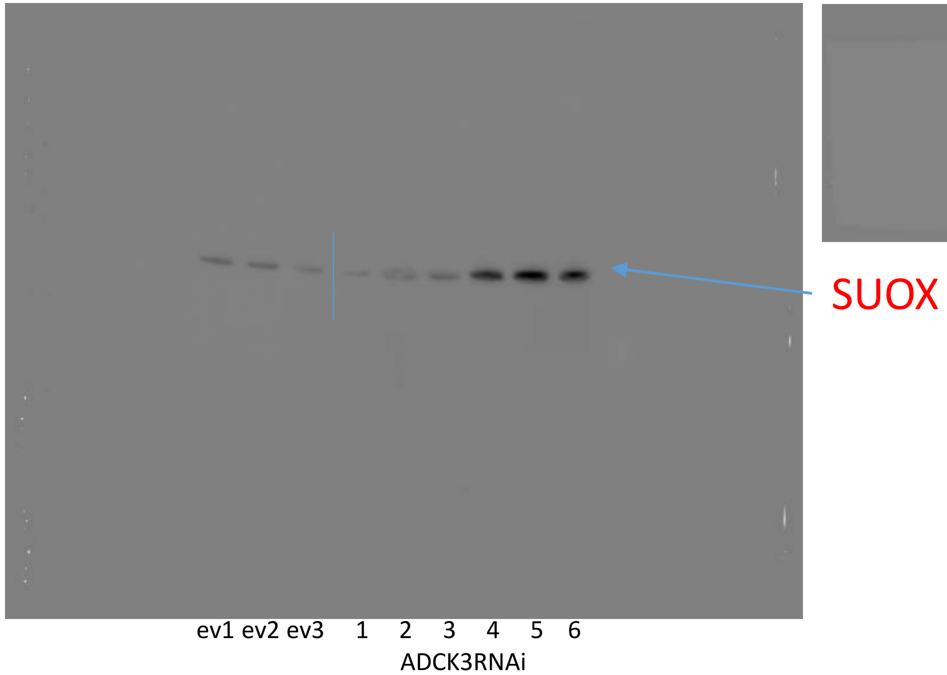

Uncut; Vinculin

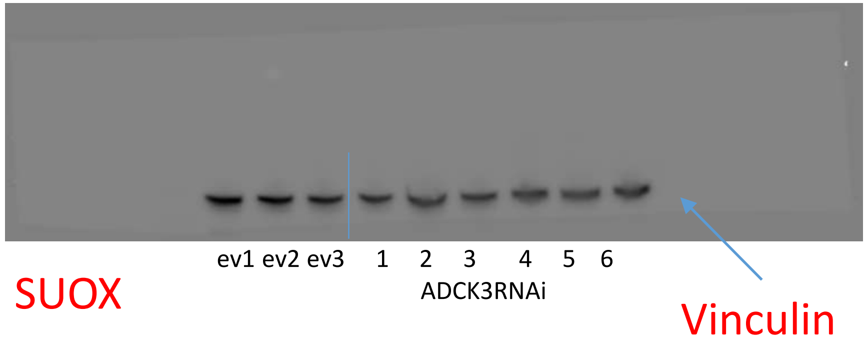

Uncut; ETHE1

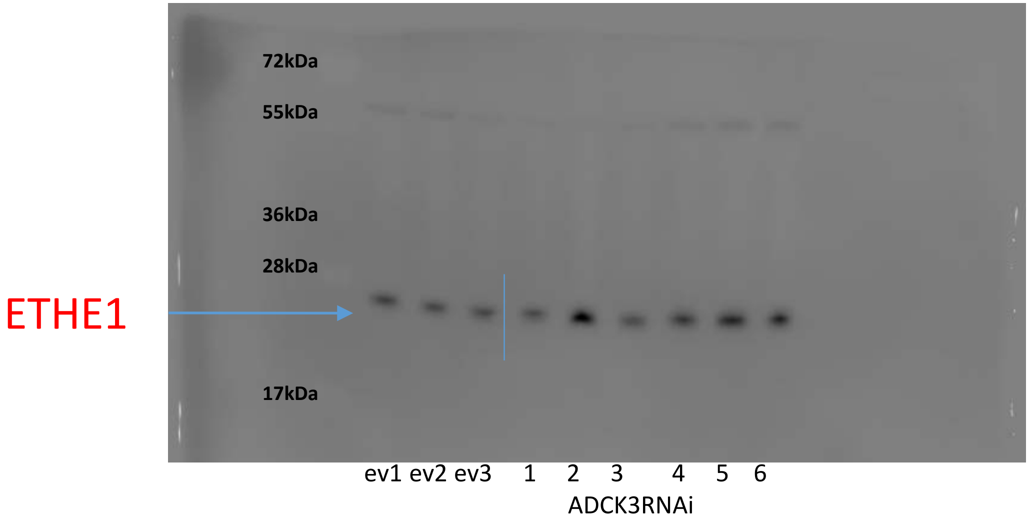

Uncut; TST

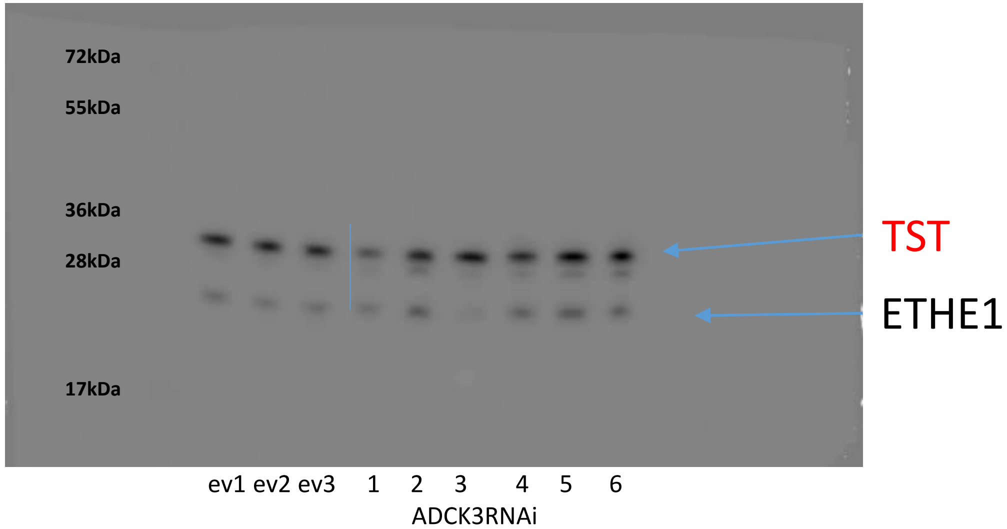

Supplement: Supplementary file 7 — Source Data for Figure 6 [file EMMM-9-96-s005.pdf]
