# Supplementary material for: Coenzyme Q deficiency causes impairment of the sulfide oxidation pathway
Source: EMBO Mol Med. 2016 Nov 17;9(1):96–111. doi: 10.15252/emmm.201606356 (PMC5210092; doi:10.15252/emmm.201606356)

SourceDataForFigure8A: Unedited membranes for SQR and TST western blots

Cut; Vinculin

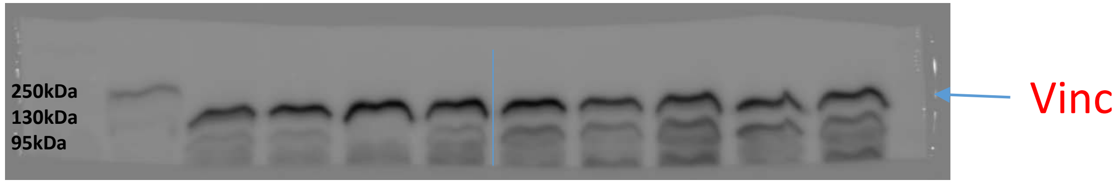


Cut; Vinculin

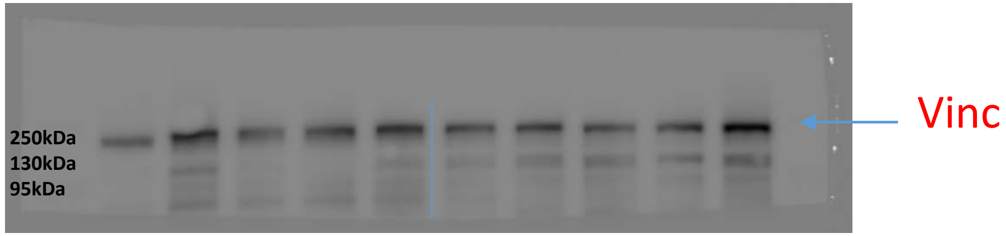


Cut; SQR

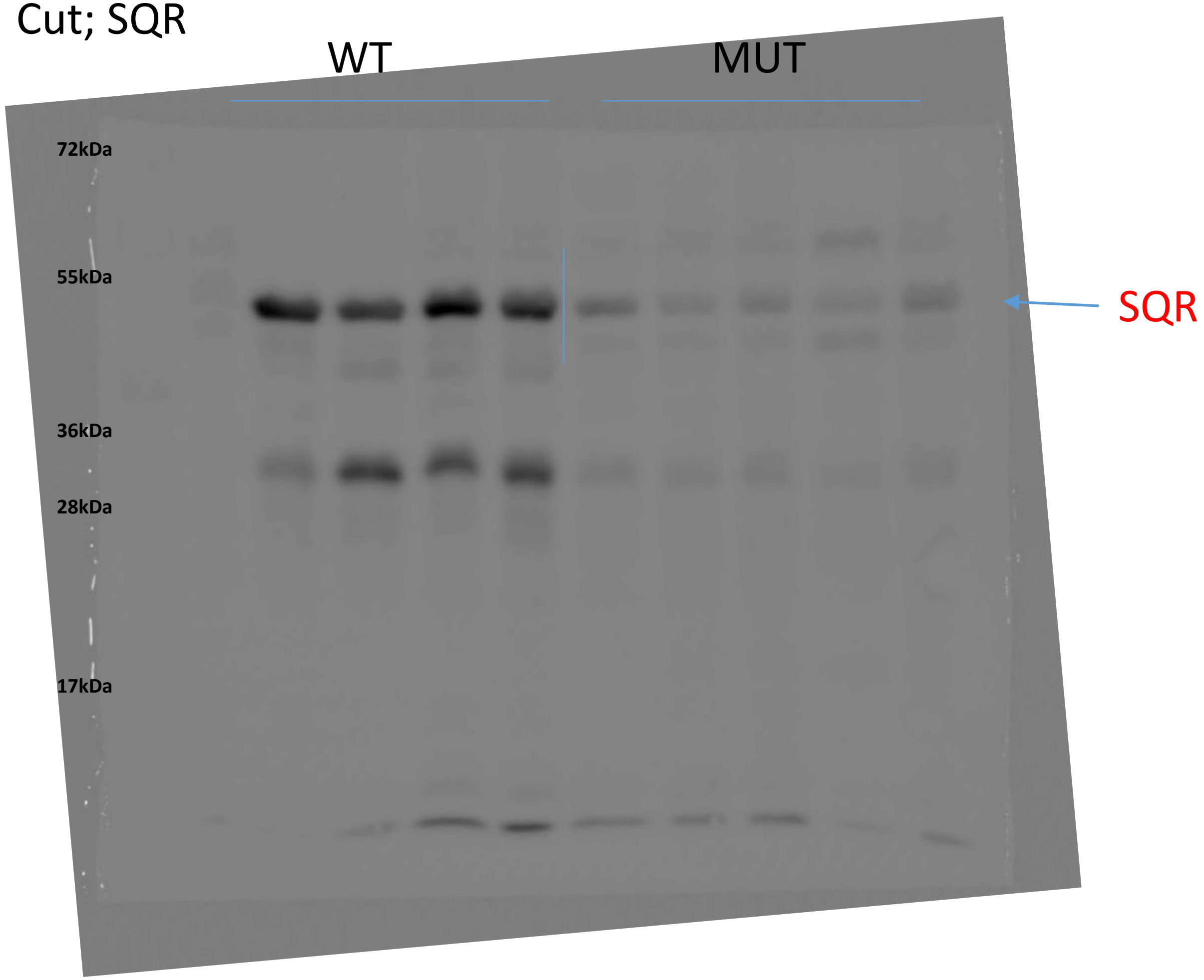


Cut; TST

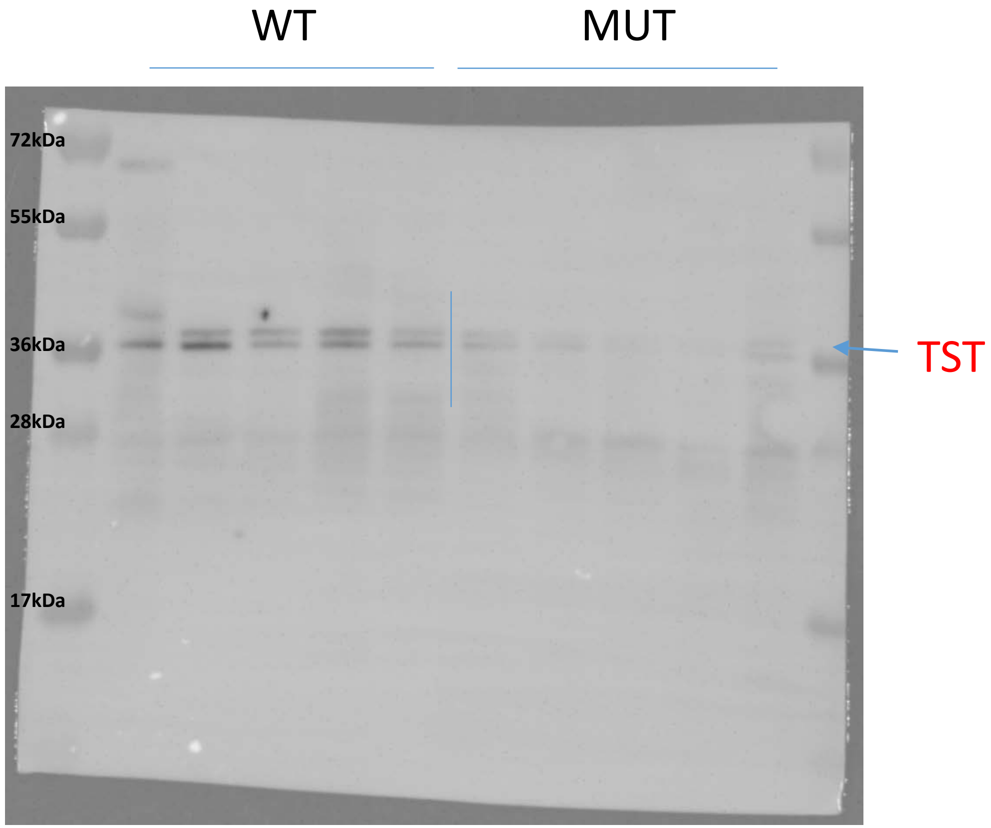

SourceDataForFigure8A: Unedited membranes for ETHE1 and SUOX western blots

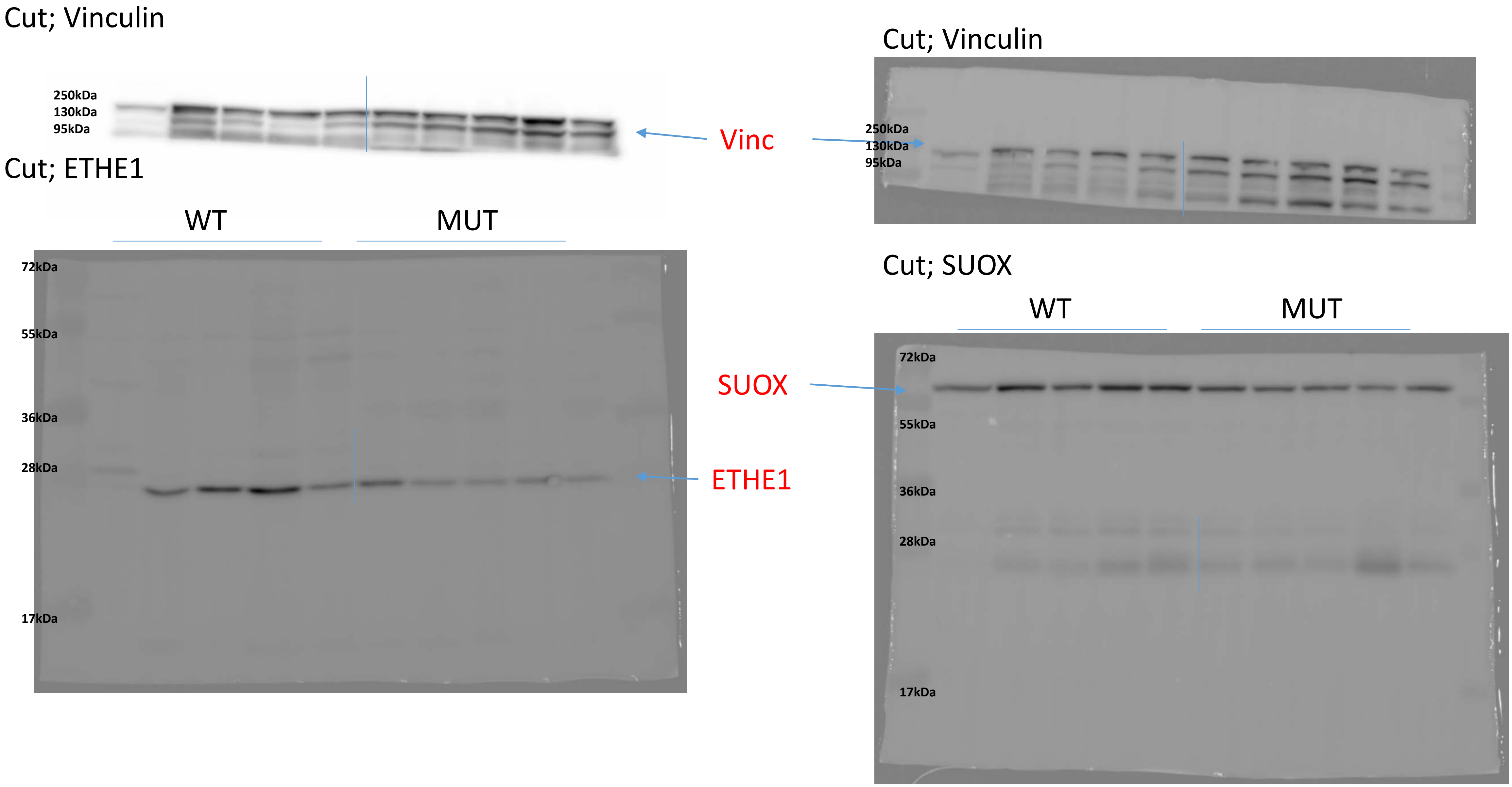

Supplement: Supplementary file 8 — Source Data for Figure 8 [file EMMM-9-96-s006.pdf]
