# Supplementary material for: Coenzyme Q deficiency causes impairment of the sulfide oxidation pathway
Source: EMBO Mol Med. 2016 Nov 17;9(1):96–111. doi: 10.15252/emmm.201606356 (PMC5210092; doi:10.15252/emmm.201606356)

SourceDataForFigure9A: Unedited membranes for SQR and TST western blots

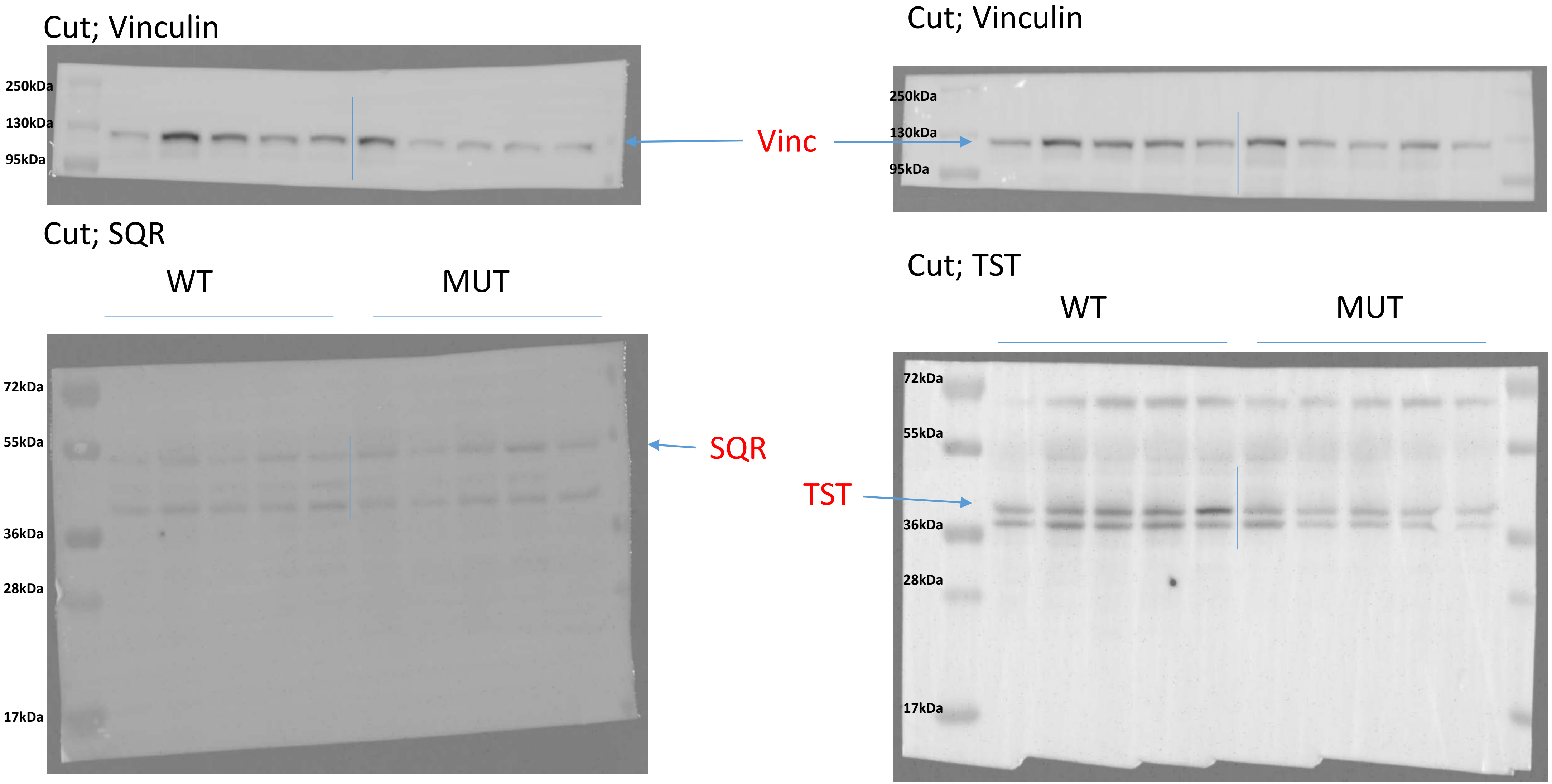

SourceDataForFigure9A: Unedited membranes for SUOX and ETHE1 western blots

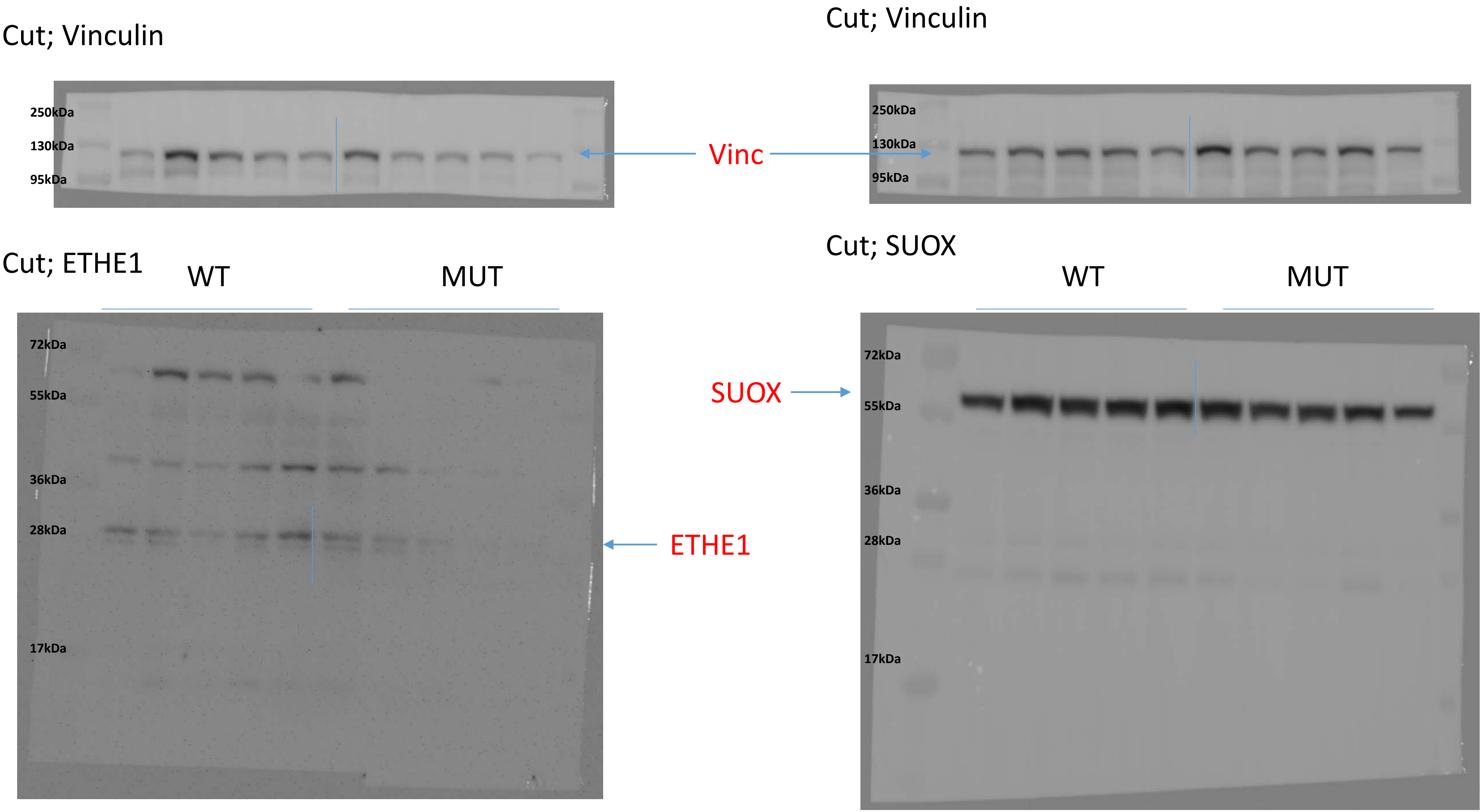

Supplement: Supplementary file 9 — Source Data for Figure 9 [file EMMM-9-96-s007.pdf]
